# Analysis of *Sogatella furcifera* proteome that interact with P10 protein of *Southern rice black-streaked dwarf virus*

**DOI:** 10.1038/srep32445

**Published:** 2016-09-22

**Authors:** Win Than, Faliang Qin, Wenwen Liu, Xifeng Wang

**Affiliations:** 1 State Key Laboratory for Biology of Plant Diseases and Insect Pests, Institute of Plant Protection, Chinese Academy of Agricultural Sciences, Beijing 100193, China

## Abstract

*Southern rice black-streaked dwarf virus* (SRBSDV) is transmitted efficiently only by white-backed planthopper (WBPH*, Sogatella furcifera*) in a persistent propagative manner. Here we used a yeast two-hybrid system to investigate the interactions between the SRBSDV- P10 and the cDNA library of WBPH. Of 130 proteins identified as putative interactors, 28 were further tested in a retransformation analysis and β-galactosidase assay to confirm the interaction. The full-length gene sequences of 5 candidate proteins: vesicle-associated membrane protein 7 (VAMP7), vesicle transport V-SNARE protein (Vti1A), growth hormone-inducible transmembrane protein (Ghitm), nascent polypeptide-associated complex subunit alpha, and ATP synthase lipid-binding protein) were amplified by 5′ rapid amplification of cDNA ends (RACE) and used in a GST fusion protein pull-down assay. Three of these proteins interacted with SRBSDV-P10 *in vitro* experiment GST pull-down assay. In a gene expression analysis of 3 different growth stages and 6 different tissue organs of *S. furcifera*, the mRNA level of VAMP7 was high in adult males and gut. Vti1A was abundant in adult female, and malpighian tubule, gut and ovary. Ghitm was predominantly found in adult male and the malpighian tubule. These research findings are greatly helpful to understand the interaction between SRBSDV and insect vector.

*Southern rice black-streaked dwarf virus* (SRBSDV), recognized as a new member of the genus *Fijivirus* in the family *Reoviridae* was first discovered in Guangdong Province, China in 2001^1^. By 2009, SRBSDV had caused disease in 19 provinces and damaged 42,000 ha in northern Vietnam and in 9 provinces and damaged more than 300,000 ha in southern China. In 2010, more than 60,000 ha of paddy fields in 29 provinces of Vietnam and more than 1,300,000 ha in 13 provinces of China became infected; in many places, crops completely failed^2^,^3^,^4^. SRBSDV is transmitted efficiently only by white-backed planthopper (WBPH, *Sogatella furcifera* Horvath, Hemiptera: Delphacidae) in a persistent propagative manner. Moreover, SRBSDV can survive on other crop plants as maize (*Zea mays*), barnyard grass (*Echinochloa crusgalli*), flaccid grass (*Pennisetum flaccidum*) and *Juncellus serotinus*^3^,^4^,^5^. During the last few years, SRBSDV has rapidly spread throughout southern China, northern Vietnam, Japan and Thailand, emerging as one of the most important rice pathogens in East and Southeast Asia due to the migration of the viruliferous WBPH vectors^3^,^4^,^6^,^7^,^8^. For WBPH nymphs and adults, the minimum virus acquisition and inoculation access periods are 5 and 30 min, respectively. The circulative transmission period of the virus in WBPH ranges from 6 to 14 days. Most viruliferous insects can transmit the virus from 2 to 6 days. In 5 days, a single insect can transmit virus to 8–25 rice plants^9^. As a typical long-distance migratory pest carried by wind currents, WBPH is distributed in most Asian counties, including China, Pakistan, Japan, Korea, Saudi Arabia, Siberia, Micronesia, Philippines, Laos, Cambodia, Myanmar, Nepal, Vietnam, Thailand, India, Indonesia, Fiji, Bangladesh and Seychelles^10^,^11^.

The viral particle of SRBSDV forms a non-enveloped icosahedral, 70–75 nm in diameter. Its 10 linear genomic segments of double-stranded RNA (dsRNA) encode seven putative nonstructural proteins (P5-1, P5-2, P6, P7-1, P7-2, P9-1, and P9-2) and six putative structural proteins (P1, P2, P3, P4, P8, and P10)^8^,^12^. The putative structural proteins are the RNA-dependent RNA polymerase (P1), major core structural protein (P2), capping enzyme (P3), outer shell B-spike protein (P4), minor core protein (P8) and major outer capsid protein (P10)^1^,^8^,^12^. The major outer capsid protein P10 of SRBSDV was used in the present study. Capsid proteins play a role in translation of viral RNA, targeting of the viral genome to its site of replication, cell-to-cell and/or systemic movement of the virus, symptomatology and virulence of the virus, activation of resistant gene-mediated host defenses, suppression of RNA silencing, interference with suppression of RNA silencing, and determination of the specificity of virus transmission by vectors^13^. The other two rice viruses such as *Rice dwarf virus* (RDV) and *Rice gall dwarf virus* (RGDV) require P2 (minor outer capsid protein) protein for virus infectivity of the vector insect^14^. In RDV, the outer capsid shell is composed of the major capsid protein P8, which plays an important role in the infection of insect vector cells^15^. Efficient virus transmission depends on specific interactions between the virus and components of the vector to allow transport of the virus in and out of insect tissues and to overcome obstacles and immune systems of insect^16^,^17^. Viruses generally have been assumed to be disseminated from the midgut into other tissues of the vector through the hemolymph^18^, but the transmission efficiency of SRBSDV by WBPH is directly associated with the level of virus in the salivary gland of the vector^19^.

Identifying the specific protein–protein interactions is essential for understanding and studying biological processes at the cellular and molecular levels^20^. The molecular mechanism and involvement of proteins in the process of virus entry, transmission, movement and replication inside the insect are largely unknown. Information on the interactions between SRBSDV and its vector *S. furcifera* is also limited. Therefore, we identified interactions between the P10 protein of SRBSDV and proteins of insect vector, *S. furcifera*. Viral movement through different organs, overcoming transmission barriers and viral replication of in the insect vector requires specific interactions between components of the virus and the insect vector^17^,^21^,^22^.

In this study, we used the yeast two-hybrid system with SRBSDV-P10 as the bait and the cDNA library of WBPH as the prey to identify protein interactions between the virus and its vector. Among 130 protein–protein interactions between SRBSDV and WBPH that were identified, vesicle-associated membrane protein 7 (VAMP7), vesicle transport V-SNARE protein (Vti1A), and growth hormone-inducible transmembrane protein (Ghitm) of WBPH were confirmed to interact with SRBSDV-P10 and to be differentially expressed in different life stages and organs of the insect. These research findings are evidence for useful information of interacted proteins between SRBSDV-P10 and vector *S. furcifera*, and the first step for further study using RNA interference (RNAi) technology to describe the functional roles of the differentially expressed proteins identified in the present study.

## Results

### Interacting proteins obtained in a yeast two-hybrid assay

**Figure 1:**
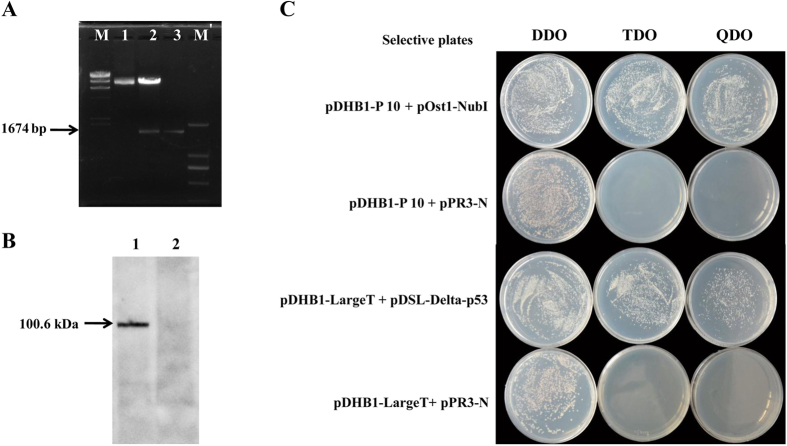
Expression verification of the bait fusion vector (pDHB1 + P10) **(A)** pDHB1 + P10 bait fusion vector confirmation by restriction enzyme digestion. Lane M: Lambda DNA/HindIII marker, lane 1: digestion of pDHB1 bait vector by enzyme, lane 2: digestion of pDHB1 + P0 bait fusion vector by enzyme, lane 3: P10, M’: DL 2,000 marker. (**B**) Detection of pDHB1 + P10 bait fusion protein in western blot. Lane 1: pDHB1 + P10, lane 2: pPR3-N empty insert (negative control). (**C**) Functional analysis of pDHB1 + P10 bait protein in yeast. Bait fusion vector (pDHB1 + P10) was used to cotransform yeast with the control plasmid pOst1-NubI or pPR3-N empty insert and grown on selective SD medium. Coexpression of P10 with Ost1-NubI resulted in growth of yeast transformants on selective media as a sign of reporter gene activation. Coexpression of P10 with pPR3-N empty insert did not yield any colonies on selective medium. pDHB1-LargeT and pDSL-p53 were used as positive controls, pDHB1-LargeT and pPR3-N-empty insert were used as negative controls.

Bait fusion vector (pDHB1 + P10) was constructed and confirmed by enzeme digestion (Fig. 1A). After confirmation, it was transformed into yeast strain NMY51. Western blot analysis with HA-tag monoclonal antibody as a probe was conducted to confirm the fusion protein of 100.6 kDa (62.6 kDa P10 + 38 kDa Cub-LexA-VP16 fusion = 100.6 kDa) in yeast cells (Fig. 1B). Moreover, in a functional analysis of the pDHB1 + P10 bait protein in yeast, we cotransformed pDHB1 + P10 bait vector with the positive control prey construct pOst1-NubI and the NubG nonsense peptide fusion vector pPR3-N as negative control prey, and then tested expression in a functional assay (Dualsystem). The yeast transformants grew on selective SD medium as a result of reporter gene activation by co-expression of the pDHB1 + P10 bait protein with pOst1-NubI. However, co-expression of pDHB1 + P10 bait protein with pPR3-N empty insert did not lead to split ubiquitin formation, and no yeast transformants grew on the selective medium (Fig. 1C). To determine optimum library screen conditions and to verify the lack of self-activation, we conducted the pilot screen analysis. Bait protein construct (pDHB1 + P10) was contransformed into yeast with the bait fusion vector pDHB1 + P10 and the empty library vector (pPR3-N-empty insert), then grown on TDO and QDO-SD medium supplemented with different concentrations of 3-aminotriazole (3-AT). No background growth was found on the selective medium with 28 mM 3-AT, so the medium was selected for the library screen (Dualsystem). The WBPH cDNA library used in this experiment was obtained from our previous study^23^. In the library screen of the pDHB1 + P10 bait and pPR3-N-WBPH cDNA prey, 482 positive colonies appeared on TDO medium supplemented with 28 mM 3-AT. After re-streaking all the positive colonies from the TDO medium onto QDO medium with 28 mM 3-AT, 441 colonies of strong interactors were then isolated and used to transform *E. coli* DH5α. From each transformation reaction, 3 colonies were picked up and checked by colony PCR analysis. Positive colonies were sent for DNA sequencing, and 339 sequences were retrieved. These sequences were used in a BLAST search of the NCBI database (http://blast.st-va.ncbi.nlm.nih.gov/Blast.cgi), and 130 interacting proteins (Table 1) were identified.

### Gene ontology (GO) annotation

**Figure 2:**
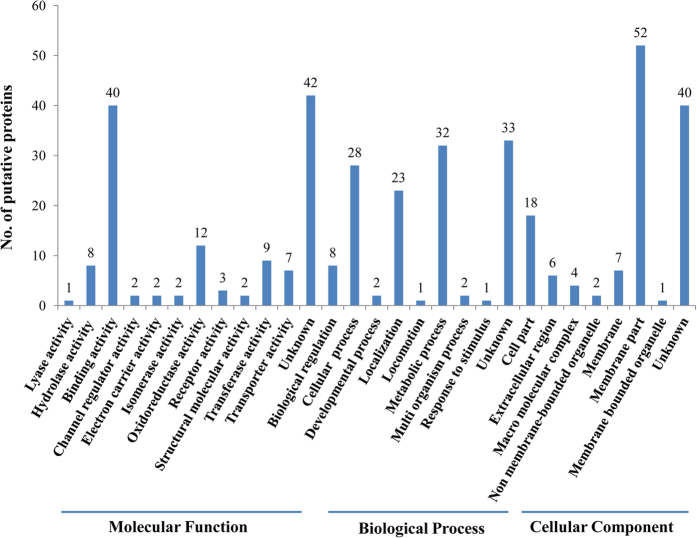
GO classification histogram of 130 WBPH putative proteins that interacted with SRBSDV-P10.

**Figure 3:**
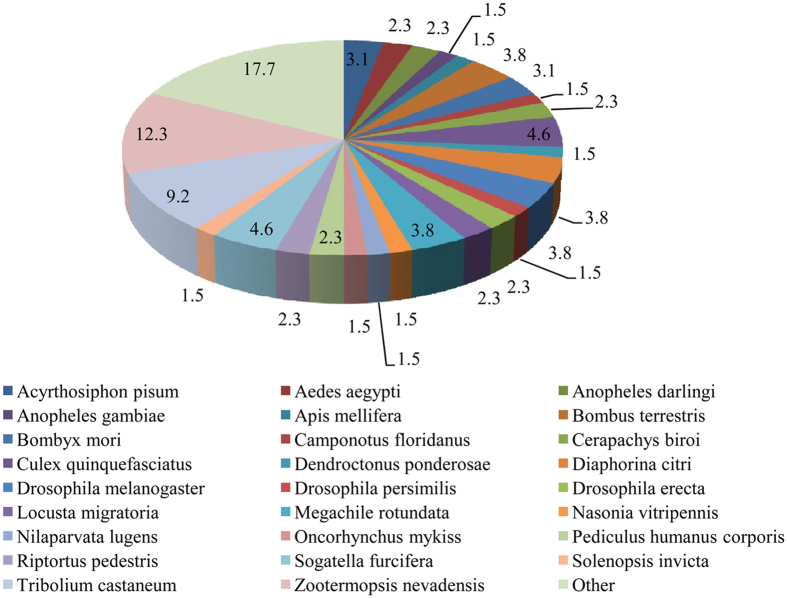
Distribution of the 130 putative proteins sequences similar to those of *Sogatella furcifera* identified from other insect species in a BLASTX search.

In the GO annotation analysis, the 130 putative interacting proteins were grouped into 12 categories of molecular function (40 proteins with binding activity, 12 with oxidoreductase activity, 9 with transferase activity, 8 with hydrolase activity, 7 with transporter activity, 3 with receptor activity, 2 with structural molecule activity, 2 channel regulatory activity, 2 with electron carrier activity, 2 with isomerase activity, 1 for lyase activity and 42 unknowns), 9 categories of biological process (32 proteins for metabolic process, 28 for cellular process, 23 for localization, 8 for biological regulation process, 2 for developmental process, 2 for multiorganism process, 1 for locomotion, 1 for response to stimulus and 33 unknowns), and 8 categories of cellular components (52 proteins in membrane parts, 18 in cell parts, 7 in membranes, 6 in extracellular region, 4 in macromolecular complex, 2 in non-membrane-bounded organelles, 1 in membrane-bounded organelle, 40 unknowns) (Fig. 2). Because the WBPH genome has not been sequenced, the proteins resulting from yeast two-hybrid assay were identified by a BLAST analysis based on their similarity with the available reference sequences, In the species distributions (Fig. 3) determined by the best match results for each sequence, 12.3% of the prey protein sequences had a maximum match with *Zootermopsis nevadensis*, followed by 9.2% with *Tribolium castanetum*, 4.6% with *S. furcifera* (WBPH) and with *Culex quinquefasciatu*, and 3.8% with *Diaphorina citri, Drosophila melanogaster, Bombus terrestris,* and *Megachile rotundata*.

### Confirmation assays: retransformation analysis and β-galactosidase assay

**Figure 4:**
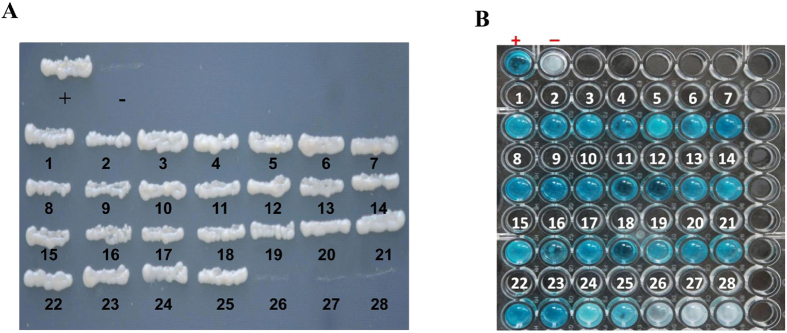
Confirmation assays: (**A**) Retransformation analysis, (**B**) β-galactosidase assay. **(A)** Each of the 28 putative interactor proteins was used with SRBSDV-P10 protein to cotransform yeast strain NMY51 cells to confirm the interaction and eliminate false positives; pDHB1-LargeT + pDSL-p53 (positive control), pDHB1-LargeT + pPR3-N-empty prey insert (negative control). (**B**) β-galactosidase assay to identify the strength of interaction between SRBSDV-P10 protein and each of the 28 putative proteins. (+) = Positive control, (−) = Negative control. (1) NADH dehydrogenase subunit 1, (2) polyubiquitin, (3) alpha tubulin 84B, (4) growth hormone-inducible transmembrane protein, (5) longwave opsin, (6) cytochrome B, (7) sugar transporter 2, (8) vesicle-associated membrane protein 7, (9) sugar transporter 6, (10) ATP synthase lipid-binding protein, (11) nascent polypeptide-associated complex subunit alpha, (12) cuticlin-1, (13) ATP citrate lyase, (14) titin, (15) glucosyl glucuronosyl transferases, (16) tetraspanin 39D, (17) coronin-1C, (18) atlastin, (19) calcium-transporting ATPase sarcoplasmic/endoplasmic reticulum type, (20) integral membrane protein 2B, (21) integrin beta-3, (22) vesicle transport V-SNARE protein Vti1A, (23) tubulin beta-2C chain, (24) cytochrome C oxidase subunit III, (25) cathepsin L, (26) carboxylesterase, (27) conserved hypothetical protein, (28) vitellogenin.

To confirm the interaction of protein-protein, we used a retransformation analysis and β-galactosidase assay. On the basis of more abundant clones, molecular function and identity percentage in the Blast results, 28 candidate plasmids (Table 2) were selected and cotransformed with the bait plasmid (pDHB1 + P10) to the yeast strain NMY51 of *Saccharomyces cerevisiae*. In the β-galactosidase assay, 15 proteins strongly interacted with SRBSDV-P10: alpha tubulin 84B, sugar transporter 2, sugar transporter 6, vesicle-associated membrane protein 7, tetraspanin 39D, cuticlin-1, atlastin, ATP synthase lipid-binding protein, cytochrome B, vesicle transport V-SNARE protein Vti1A, NADH dehydrogenase subunit 1, nascent polypeptide-associated complex subunit alpha, polyubiquitin, growth hormone-inducible transmembrane protein, and tubulin beta-2C chain. Ten other proteins interacted weakly with SRBSDV-P10: longwave opsin, ATP citrate lyase, titin, glucosyl glucuronosyl transferases, coronin-1C, calcium-transporting ATPase sarcoplasmic/endoplasmic reticulum type, integral membrane protein 2B, integrin beta-3, cytochrome C oxidase subunit III, cathepsin L. Three proteins did not interact with SRBSDV-P10: carboxylesterase, conserved hypothetical protein, and vitellogenin (Fig. 4).

### Full-length gene amplification

For testing a protein–protein interaction in a pull-down assay, the full length of the gene sequence is needed. Based on the available gene fragment from the yeast two-hybrid screening result, strongly interacting proteins in the retransformation analysis and β-galactosidase assay, the vesicle-associated membrane protein 7, vesicle transport V-SNARE protein Vti1A, growth hormone-inducible transmembrane protein, nascent polypeptide-associated complex subunit alpha, and ATP synthase lipid-binding protein were selected and amplified, and the full-length sequence was obtained using 5′ rapid amplification of cDNA ends (RACE). Full-length gene sequences for 5 selected proteins could be amplified; the full-length of the genes, including the 5′-untranslated region (5′ UTR), open reading frame (ORF), 3′-untranslated region (3′ UTR), and poly (A) tail of each gene are described in Supplemental Table S1.

### Analysis of protein sequence alignments

#### Vesicle-associated membrane protein 7 (VAMP7)

Sequence alignment analysis showed that vesicle-associated membrane protein 7 (VAMP7) of *S. furcifera* had 73% identity to that of *Athalia rosae* (Hymenoptera), 69% identity to *Tribolium castaneum* (Coleoptera), 67% to *Halyomorpha halys* (Hemiptera), and 58% to *Drosophila melanogaster* (Diptera).

#### Vesicle transport V-SNARE protein (Vti1A)

Protein sequence alignment result revealed that vesicle transport V-SNARE protein (Vti1A) of *S. furcifera* had 59% identity to *Cimex lectularius* and 56% to *Halyomorpha halys* (Hemiptera), 55% to *Camponotus floridanus* and 54% to *Dinoponera quadriceps* (Hymenoptera), and 50% to *Pediculus humanus corporis* (Phthiraptera).

#### Growth hormone-inducible transmembrane protein

This protein sequence of *S. furcifera* had 61% identity to that of *Oryctes borbonicus* (Coleoptera), 59% identity to *Halyomorpha halys* (Hemiptera), 55% to *Pogonomyrmex barbatus*, and *Megachile rotundata* (Hymenoptera), and 52% to *Pediculus humanus corporis* (Phthiraptera).

#### Nascent polypeptide-associated complex subunit alpha

Sequence alignment showed that nascent polypeptide-associated complex subunit alpha of *S. furcifera* had 74% to *Bombus impatien* and *Apis dorsata* (Hymenoptera), 65% to *Bombyx mori* (Lepidoptera), and 63% to *Drosophila melanogaster* (Diptera).

#### ATP synthase lipid-binding protein

Protein sequence alignment showed that this protein of *S. furcifera* had 82% identity to *Halyomorpha halys* (Hemiptera), 78% to *Acromyrmex echinatior* and *Dinoponera quadriceps*(Hymenoptera), 78% to *Tribolium castaneum* (Coleoptera), and 75% to *Drosophila melanogaster*) (Diptera).

#### GST pull-down assay to verify the proteins that interacted with SRBSDV-P10

**Figure 5:**
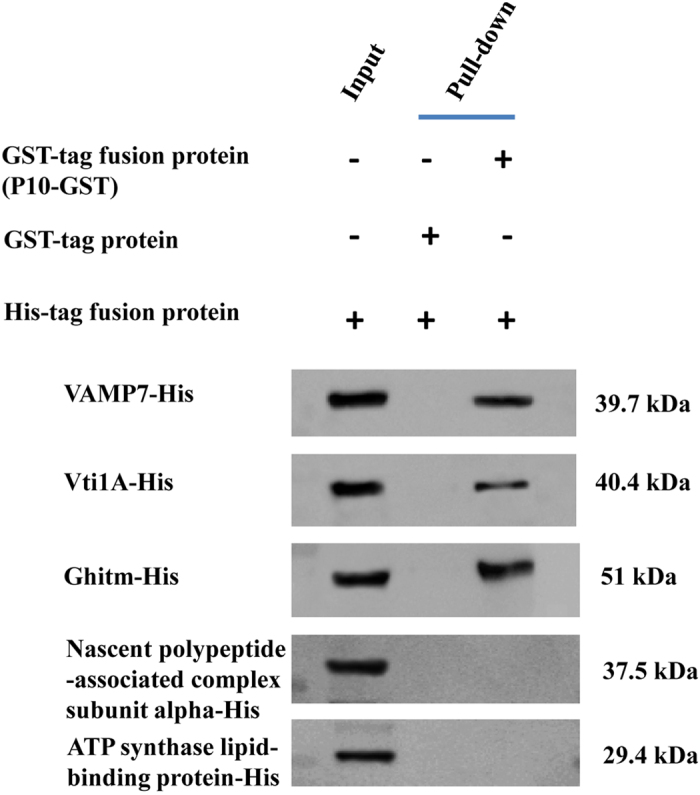
GST pull-down assay of the interaction between SRBSDV-P10 and WBPH proteins and confirmation by Western blot. Lane 1, His-tag fusion protein (Protein-His) as input; lane 2, GST protein with His-tag fusion protein (negative control); lane 3, GST fusion protein (P10-GST) with His-tag fusion protein. The size of the His tag fusion protein for VAMP7 was (24.7 kDa + 15 kDa) 39.7 kDa, the His tag fusion protein for Vti1A was (25.4 kDa + 15 kDa) 40.4 kDa, the His tag fusion protein for Ghitm was (36 kDa + 15 kDa) 51 kDa, the His tag fusion protein for nascent polypeptide-associated complex subunit alpha was (22.5 kDa + 15 kDa) 37.5 kDa, and the His tag fusion protein for ATP synthase lipid-binding protein was (14.4 kDa + 15 kDa) 29.4 kDa.

**Figure 6:**
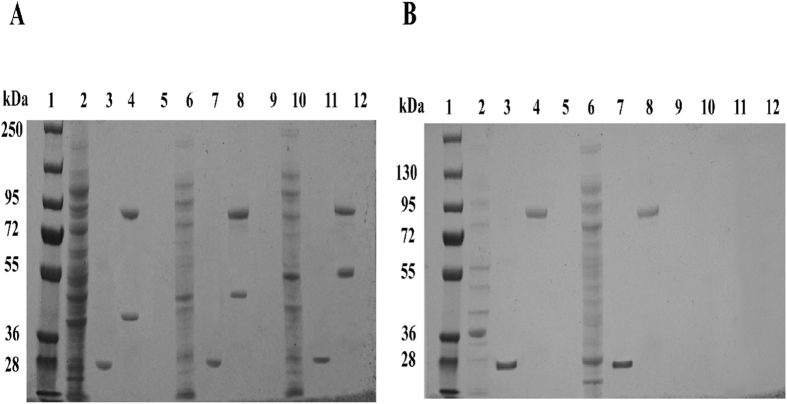
GST pull-down analysis of the interaction between SRBSDV-P10 and WBPH proteins by by SDS-PAGE. **(A)** Lane 1: Protein ladder. 2: VAMP7-His (39.7 kDa) (Input). 3: VAMP7-His + GST (Pull-down = Negative control) (GST = 27 kDa). 4: VAMP7-His + P10-GST (Pull-down) (P10-GST = 89.64 kDa) (VAMP7-His = 39.7 kDa). 5: Free lane. 6: Vti1A-His (40.4 kDa) (Input). 7: Vti1A-His + GST (Pull-down = Negative control) (GST = 27 kDa). 8: Vti1A-His + P10-GST (Pull-down) (P10-GST = 89.64 kDa) (Vti1A -His = 40.4 kDa). 9: Free lane. 10: Ghitm-His (51 kDa) (Input). 11: Ghitm-His + GST (Pull-down = Negative control) (GST = 27 kDa). 12: Ghitm-His + P-10-GST (Pull-down) (P10-GST = 89.64 kDa) (Ghitm-His = 51 kDa). **(B)** Lane 1: Protein ladder. 2: Nascent polypeptide-associated complex subunit alpha-His (37.5 kDa) (Input). 3: Nascent polypeptide-associated complex subunit alpha-His + GST (Pull-down = Negative control) (GST = 27 kDa). 4: Nascent polypeptide-associated complex subunit alpha-His + P10-GST (Pull-down) (P10-GST = 89.64 kDa). 5: Free lane. 6: ATP synthase lipid-binding protein-His (29.4 kDa) (Input). 7: ATP synthase lipid-binding protein-His + GST (Pull-down = Negative control) (GST = 27 kDa). 8: ATP synthase lipid-binding protein-His + P10-GST (Pull-down) (P10-GST = 89.64 kDa).

In the GST pull-down assay for the 5 WBPH proteins: VAMP7, Vti1A, Ghitm, nascent polypeptide-associated complex subunit alpha, and ATP synthase lipid-binding protein, 3 were confirmed to interact with SRBSDV-P10: VAMP7, Vti1A, and Ghitm of WBPH. The results of the GST pull-down assay were then analysed by Western blot (Fig. 5 and Supplemental Fig. 1A,B) and SDS-PAGE (Fig. 6). As a result of the pull-down assay using the combination of the GST-tag fusion protein (P10-GST) with the His-tag fusion protein for VAMP7, Vti1a and Ghitm were shown to interact using a Western blot and SDS-PAGE analysis, and nascent polypeptide-associated complex subunit alpha, and ATP synthase lipid-binding protein did not. In contrast, in the control pull-down assay, none of the combinations of the GST fusion protein (GST alone) with the His-tag fusion protein interacted in the analyses.

#### Gene expression in different growth stages and organs of *Sogatella furcifera*

**Figure 7:**
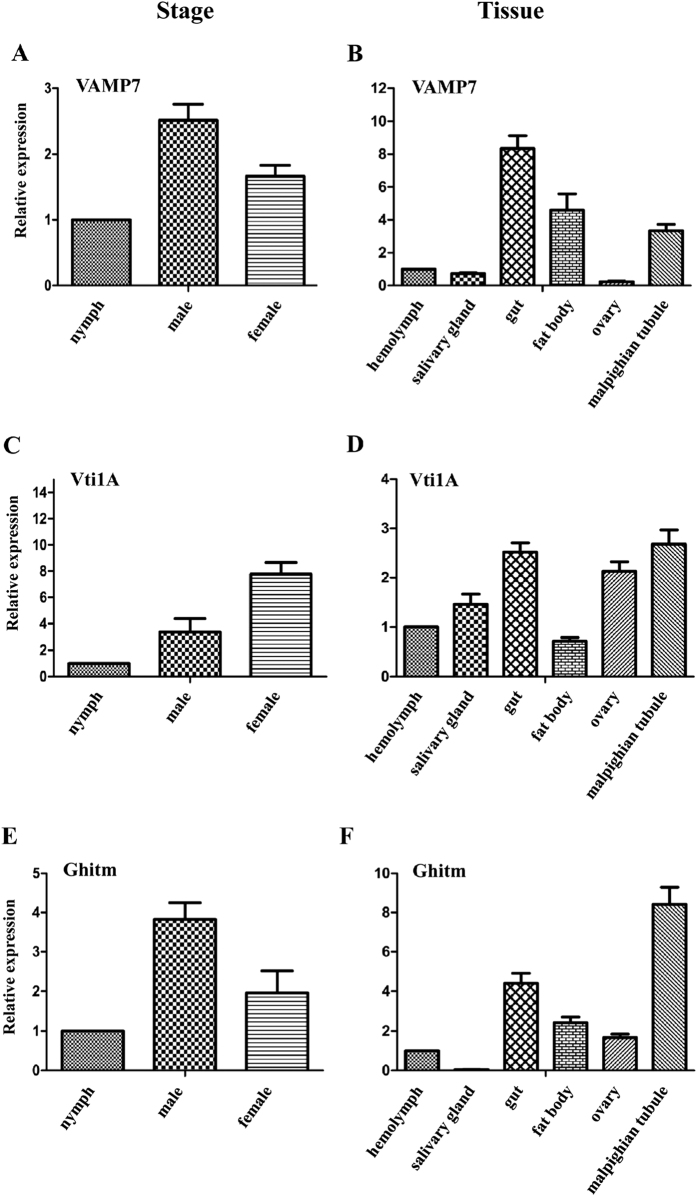
Relative mRNA transcript levels for 3 candidate proteins in different growth stages (nymphs, male adults, female adults) and in different organs (hemolymph, salivary gland, gut, fat body, ovary, malpighian tubule) of *Sogatella furcifera* by RT-qPCR. (**A**,**B**) Vesicle-associated membrane protein 7 (VAMP7). (**C**,**D**) Vesicle transport V-SNARE protein (Vti1A). (**E**,**F**) Growth hormone-inducible transmembrane protein (Ghitm).

The relative quantity of the mRNA transcript levels of the three candidate genes (VAMP7, Vti1A, and Ghitm) in WBPH at 3 growth stages (nymphs, adult males and adult females) and 6 organs (hemolymph, salivary gland, gut, malpighian tubule, ovary and fat body) was then analysed using a comparative ΔΔCt method. The mRNA level of VAMP7 and Ghitm was high in adult males, followed by adult females and nymphs (Fig. 7). The mRNA level of Vti1A was abundant in female adults, followed by adult males and nymphs. In the 6 tissue organs, the highest mRNA transcript level for VAMP7 was found in the gut followed by the fat body, then the malpighian tubule, hemolymph, and salivary gland; expression in the ovary was negligible. Vti1A was highly expressed in the malpighian tubule followed by the gut, ovary, salivary gland, hemolymph and fat body. Ghitm was predominantly found in the malpighian tubule followed by the gut, then the fat body, ovary, and hemolymph, with negligible expression in the salivary gland.

## Discussion

In this experiment, major outer capsid protein P10 of SRBSDV as a bait protein was used to screen the cDNA library of insect vector *Sogatella furcifera* and 130 putative interacting proteins were identified. Three proteins of *S. furcifera*—vesicle-associated membrane protein 7 (VAMP7), vesicle transport V-SNARE protein (Vti1A), and growth hormone-inducible transmembrane protein (Ghitm)—interacted with SRBSDV-P10 and were differentially expressed in nymphs, adult males and females and different organs of WBPH. Although we still need to elucidate how the three proteins are involved in the molecular interaction with the virus, they were shown to interact with the virus in an *in vitro* experiment.

On the basis of the GO annotation analysis, VAMP7 is a member of the SNARE (soluble N-ethylmaleimidesensitive fusion protein-attachment protein receptor) super family, which have SNARE binding activity and are involved in vesicle-mediated transport. The SNARE complex mediates membrane fusion, important for trafficking of newly synthesized proteins, recycling of pre-existing proteins and organelle formation. Vesicle-associated membrane proteins (VAMPs) are required for the trafficking of vesicles between membrane-bound intracytoplasmic organelles and are also important in the facilitation of neurosecretion and in constitutive and regulated secretion in non-neuronal cells^24^. VAMP7 might thus facilitate virus release from the vesicle during trafficking with the aid of Vti1A, which is located in the membranes of target vesicle compartments. If each distinct trafficking step requires specific SNAREs or a particular subset of SNAREs, many other VAMPs and syntaxins are likely to function within the endosomal network^25^. In our RT-qPCR result, VAMP7 was found most abundantly in the gut and was second least abundant in the salivary gland. VAMP7 defines a novel membrane compartment in neurite outgrowths and in the somatodendritic domain^26^ and is involved in a trafficking pathway that is common to most eukaryotic cell types^27^. VAMP7, localized primarily in lysosomal compartments in many cell types, has a well-established role in lysosomal delivery^28^,^29^. The apical plasma membrane of polarized intestinal cells of humans was also enriched in VAMP7, which also has been characterized as a vesicle SNARE involved in the delivery of membrane type 1-matrix metalloproteinase (MT1-MMP) to invadopodia^25^,^30^. The existence of numerous SNARE-related proteins, each apparently specific for a single kind of vesicle or target membrane, indicates that NSF and SNAPs may be universal components of a vesicle fusion apparatus common to both constitutive and regulated fusions (including neurotransmitter release), for which the SNAREs may help to ensure vesicle-to-target specificity. VAMP7 is involved in transport to lysosomes during lysosomal secretion in fibroblasts^27^,^30^.

Vesicle transport V-SNARE protein (Vti1A), shown by RT-qPCR to be primarily in the malpighian tubule, gut and the ovary of WBPH, is also a member of the SNARE super family and functions in intracellular trafficking. Vesicle trafficking between intracellular compartments of eukaryotic cells is mediated by conserved protein machineries. In each trafficking step, fusion of the vesicle with the acceptor membrane is driven by a set of distinctive SNARE proteins^31^.

Vti1A is located in the membranes of target vesicle compartments. Vti1A and VAMP7 belong to the SNARE complex^32^. Vti1A and SNARE protein Vti1B were knocked down by RNA interference, resulting in a significant decrease in the mass of engorged ticks compared with the controls during prolonged blood-feeding on the host^33^. RNA interference of either Vti1A or Vti1B impaired oviposition, and none of the ticks produced eggs masses. These results show an important functional role of the Vti family of SNARE proteins in tick blood-feeding and ultimately in oviposition In HeLa and Neuro2A cells, siRNA (small interfering RNA)-mediated knockdown of Vti1A or VAMP7 inhibited trafficking of K^+^ channel-interacting proteins Kv4/KChIP1 to the plasma membrane^32^.

Growth hormone-inducible transmembrane protein (Ghitm) was found mainly in the malpighian tubule, and in the GO analysis was annotated as having protein binding activity. Ghitm is a mitochondrial protein and a member of the transmembrane BAX inhibitor motif (TMBIM) family and BAX inhibitor-1 (BI1) super family. Different studies have indicated that all TMBIM family members have inhibitory activities in apoptosis^34^. This protein localizes specifically to the inner mitochondrial membrane IMM, where it regulates apoptosis through two separate processes: (1) the BAX-independent management of mitochondrial morphology and (2) the release of cytochrome *c*. Ghitm is also involved in the morphology of specific cristae structures in the mitochondria, controls the release of cytochrome *c* from the mitochondria, and can potentially interfere with apoptosis to promote cell survival. It may also play a role in apoptosis through maintaining calcium ion homeostasis in the endoplasmic reticulum (ER)^35^,^36^. On the basis of structural comparisons among members of the BI1 family, Ghitm may be a regulator of cell death pathways^37^.

Although the other interacting proteins identified by the retransformation analysis and β-galactosidase assay still need to be confirmed by other appropriate methods, we briefly describe their function here. The alpha subunit is one of two subunits of the nascent polypeptide-associated complex (NAC) and contributes to the prevention of inappropriate interactions^38^. NAC mutations cause severe embryonic lethal phenotypes in mice, *Drosophila melanogaster*, and *Caenorhabditis elegans*^39^. The nascent polypeptide-associated complex (NAC) is a key regulator of proteostasis to provide the cell with a regulatory feedback mechanism in which translational activity is also controlled by the folding state of the cellular proteome and the cellular response to stress^40^.

ATP synthase lipid-binding protein in the mitochondria catalyzes ATP synthesis, utilizing an electrochemical gradient of protons across the inner membrane during oxidative phosphorylation^41^. Beta-tubulin is a subunit of tubulin, one of several members of a small family of globular proteins^42^. Tubulins are major components of the microtubules that are the main structures for functions such as the spatial distribution of organelles, cell motility, and intracellular transport in eukaryotic organisms. Modifications of tubulin play a vital role for regulating microtubule properties, such as structure and stability of microtubule, microtubule-based functions including ciliary beating, cell division and intercellular trafficking^43^,^44^.

Sugar transporters are essential for controlling carbohydrate transport in numerous organisms from bacteria to mammals and mediate the movement of sugars into cells^45^. Tetraspanins function as organizers of the cell surface by recruiting specific partner proteins into tetraspanin-enriched microdomains, which regulate processes such as cell adhesion, signalling and intracellular trafficking^46^. Isoforms 84B and 85E of alpha tubulin (*Drosophila*) have distinct functions, suggesting that isoform specialization plays a key role in the control of microtubule assembly in complex metazoans^47^. Atlastins are integral membrane proteins with a large N-terminal cytosolic region containing the GTPase domain, two transmembrane domains and a cytosolic C-terminus of variable length^48^. Cytochrome *b*, in the mitochondria of eukaryotic cells, is part of the electron transport chain and is the main subunit of the transmembrane cytochrome bc1 and b6f complexes^49^,^50^. NADH dehydrogenase subunit 1 is involved in the first step of the electron transport chain of oxidative phosphorylation. Alterations in the electron transport components by mutations in mtDNA may compromise the normal electron flow and lead to an increase of bifurcation and generation of superoxidase radicals and increase oxidative stress in various types of cancer cells^51^.

The cuticle of insects is a multi-functional exoskeleton and highly impervious barrier between the animal and its environment. Essential for maintaining body morphology and integrity, it also has a critical role in locomotion via attachments to body-wall muscles^52^. Their composite structural materials confer mechanical properties that are optimal for their biological functions and to a large extent determined by the interactions between various cuticular components, mainly the chitin filament system and the proteins^53^. According to a proteomic analysis, several cuticle proteins were also differentially expressed in genotypes of *S. graminum* that differed in their ability to transmit CYDV-RPV2^54^. When the gene encoding the cuticle protein is silenced by RNAi, the number of viral particles of the virus in the vector insects decreased, and virus transmission efficiency by the vector then declined^55^. The cuticular protein of *Rhopalosiphum padi* interacted with readthrough protein (RTP) of *Barley yellow dwarf virus*-GPV (BYDV-GPV) and was differentially expressed between viruliferous and healthy aphids^56^.

Ubiquitin (Ub) protein, found in almost all tissues of eukaryotic organisms, is important in numerous diverse cellular functions, including protein degradation by the proteasome, immune signaling, cell cycling, lysosomal degradation, autophagy, apoptosis, endocytosis, and ER-associated degradation^57^. The ubiquitin proteasome system (UPS) has a prominent influence on virus–host interactions at almost every stage of antiviral defense in plants and animals^58^.

Understanding the mechanisms by which virus and receptor interactions induce signaling pathways and how these interactions might actively mediate internalization of the virus/receptor complex is very limited^59^. In our gene expression analysis of different growth stages and different organs of *S. furcifera*, the mRNA level of 3 candidate proteins was greater in male and female adults than in nymphs. Although mRNA transcripts of VAMP7 was mainly found in the gut and Ghitm transcripts were most abundant in the malpighian tubule, Vti1a mRNA was highest in the gut, ovary and malpighian tubule.

Taken together, our results demonstrated the *in vitro* interaction of 3 candidate proteins of WBPH with the P10 protein of SRBSDV, but their interaction *in vivo* still needs to be demonstrated. To investigate how those proteins actually interact with SRBSDV in these tissues and influence viral transmission, we will study the functional role of these proteins using RNAi and verify the identity and involvement of the proteins in virus transmission using confocal microscopy assays. Our findings provide a basis for functional analyses of the proteins, and their interaction between SRBSDV and insect vector and for elucidating their roles in the largely unknown molecular mechanisms underlying virus entry, movement and replication inside the insect and viral transmission into new hosts.

## Methods

### Virus maintenance and insect rearing

Laboratory isolate of SRBSDV (GenBank accession JQ773429) was originally collected from Guangzhou, China, and virus-infected leaves were kept at −70 °C. SRBSDV-infected rice plants with typical symptoms were kept and grown in a greenhouse. Nonviruliferous WBPH (*S. furcifera*), originally captured in Nanjing, China, were reared in glass beakers with rice seedlings in a laboratory incubator at 26 °C with 16 h light/8 h dark. The insects were transferred onto fresh rice seedlings every week^60^.

### Bait fusion vector (pDHB1 + P10) construction

The bait fusion vector (pDHB1 + P10) plasmid was constructed by cloning the full-length SRBSDV-P10 gene (GenBank accession JQ773429) using specifically designed primers, and it was then inserted into LexA-pDHB1. The resulting bait plasmid pDHB1 + P10 was used to transform yeast (*Saccharomyces cerevisiae*) strain NMY51. Positive transformants were selected on minimal synthetic defined medium lacking leucine (SD/−Leu). Expression of the bait construct pDHB1 + P10 was verified by Western blotting using HA-tag monoclonal antibody. A functional assay was done using an autoactivation analysis with a DUALhunter starter kit (Dualsystems Biotech, Zurich, Switzerland) according to the manufacturer’s protocol.

### cDNA library of Sogatella furcifera

The WBPH cDNA library used in this experiment was obtained from our previous study^23^.

### Library screening using a yeast two-hybrid assay

The yeast two-hybrid assay to examine interactions of proteins between SRBSDV- P10 and its insect vector WBPH was done using a DUALhunter starter kit (Dualsystems Biotech) based on split-ubiquitin. The pDHB1 + P10 fusion vector and *S. furcifera* cDNA library were used to cotransform yeast strain NMY51 by the lithium acetate method with single-stranded nucleic acids (ssDNA) as the carrier according to the manufacturer’s protocol. Selected triple dropout medium (TDO: S.D./-His/-Leu/-Trp) supplemented with 3-aminotriazole (3-AT) was used to select positive-interacting clones. Positive clones were restreaked on selected quadruple dropout medium (QDO: S.D./-Ade/-His/-Leu/-Trp) supplemented with 3-AT. The strength of the protein–protein interaction between the bait and prey expressed in a particular clone was evaluated in a β-galactosidase assay using the HTX High-throughput β-Galactosidase Assay Kit (Dualsystems Biotech).

### Gene Ontology (GO) annotations of positive interactors

Plasmids were recovered from the positive interactors and inserted into *E. coli* strain DH5-α, which was then cultured on LB agar plates supplemented with 100 mg/mL ampicillin. Colonies were checked by PCR and sent for sequencing to determine the putative insect proteins that interacted with SRBSDV-P10. The resultant sequences were assembled by the SeqMan II program (DNAstar) to exclude duplicated sequences. The different sequences were then used as queries in a BLAST search of the NCBI database (http://blast.st-va.ncbi.nlm.nih.gov/Blast.cgi), and the tentative interacting proteins were identified and then annotated using Gene Ontology (GO) for molecular function, biological process and cellular component using the UniProt KB database (http://www.uniprot.org/).

### Retransformation analysis and β-galactosidase assay

Each of the 28 selected positive library plasmids of the putative interacting proteins were inserted into yeast strain NMY51 of *Saccharomyces cerevisiae* with the bait plasmid (pDHB1 + P10) or with the positive and negative control plasmids, then grown on DDO and QDO supplemented with 3-AT. The HTX High-throughput β-Galactosidase Assay Kit (Dualsystems Biotech) β-galactosidase assay was used to distinguish false-positive interactions and strong interactors.

### Full-length gene amplification for selected proteins

The nucleotide sequences resulting from the yeast two-hybrid assay were not complete sequences for the genes. To obtain the full-length gene sequence needed for the pull-down assay, the full-length gene was amplifid using the 5′ RACE system kit (Invitrogen, Carlsbad, CA, USA) according to the manufacturer’s instruction. Total RNA from WHPB was extracted using the standard Trizol reagent protocol (Invitrogen). Gene-specific primers (GSP1, GSP2, GSP3) were designed for the full-length gene amplification. After the final PCR using primer GSP-3, the PCR product was separated by 1% agarose gel electrophoresis and purified using the AxyPrep DNA Gel Extraction Kit (Axygen, Biosciences, USA). The gel-purified product was then cloned into the pMD19-T simple vector (Takara, Japan) and sent for sequencing. The resultant sequence was identified by using Vector NTI software (Life Technology, Invitrogen) to reveal the open reading frame (ORF), the presumed amino acid sequence, 5′ and 3′ untranslated regions, and molecular weight of the deduced amino acid sequence. The standard protein–protein BLAST sequence comparison and PSI-BLAST programs (http://blast.ncbi.nlm.nih.gov/) were used to search for sequences in the GenBank- and Swiss-Prot databases with similarities to the translated protein of each genes.

### GST pull-down assay

The full-length SRBSDV-P10 gene sequence (Gen Bank accession JQ773429) was obtained from virus-infected rice plants by using specifically designed primers. The cDNA fragments were amplified and cloned into pGEX-6p-1 for fusion with glutathione *S*-transferase (GST) as the bait. Full-length sequences of the genes obtained by the 5′ RACE system were obtained from WBPH and amplified by using reverse transcription polymerase chain reaction (RT-PCR) with the aid of the designed primer and inserted into pCOLD-SUMO as the prey for fusion with His-tag. Both bait and prey recombinant proteins were used to transform *E. coli* strain BL-21 for protein expression. The GST pull-down assay was done using the Pierce GST Protein Interaction Pull-Down Kit (Pierce Biotechnology, Rockford, Illinois USA) according to the manufacturer’s protocol. The GST fusion protein (P10-GST) was immobilized on a Pierce Spin Column tube containing glutathione agarose resin by gentle rocking motion on a rotating platform for 3 h incubated at 4 °C. Then, the spin column tube with the mixture was centrifuged at 1250 × *g* for 1 min and washed five times with a wash solution (1:1 TBS to Pull-Down Lysis Buffer). The His-tag fusion protein was added to the spin column tube, and after a3-h of gentle rocking motion on a rotating platform at 4 °C, the tube was centrifuged at 1250 × *g* for 1 min and washed five times with the wash solution. The bait and prey bounded proteins were separated by SDS-PAGE gel electrophoresis and detected by Western blotting with His-tag antibodies. Here, the combination of GST protein (GST alone) with His-tag fusion protein was used as a negative control.

### RT-qPCR to quantify relative mRNA transcript levels

#### Sample preparations for gene expression

The expression of each gene at different life stages and tissues in WBPH was analysed using 3 pools of 20 insects for every stages and 3 pools of 50 adults for various tissues. Each pooled sample was placed in a 1.5 ml microcentrifuge tube and immediately frozen with liquidnitrogen and stored at −80 °C. For tissue sample preparation, 50 fifth-instar nymphs were anesthetized for 5 min on ice, then each was placed on a glass slide to separate the head–prothorax from the mesothorax using a stereomicroscope. Drops of hemolymph were quickly collected from the exposed end of the mesothorax. The salivary glands, gut, malpighian tubules, ovary, and fat body were then dissected in diethylpyrocarbonate (DEPC)-treated water using fine forceps and placed into separate 1.5 ml microcentrifuge tubes containing 200 μl of DEPC-treated water. The DEPC-treated water was then replaced with 200 μl of Trizol reagent. Samples were homogenized using a small pestle and stored at −80 °C until use. Tissue samples were prepared in triplicate.

#### Sample preparations for gene expression

According to the standard Trizol reagent protocol (Invitrogen), total RNA was extracted from the WBPH samples according to the manufacturer’s instructions. The purity and amount of extracted RNA were quantified using a Nanodrop 2000 ultraviolet spectrophotometer (Thermo Scientific, Wilmington, DE, USA) as the ratio of OD260/OD280. cDNA was synthesized from 1 μg of total RNA using a Fast Quant RT Kit (TIANGEN, Beijing, China). For internal reference, the 18S rRNA gene was used to normalize the quantity of total RNA purified from each sample. The PCR protocol was conducted using a Super Real PreMix Plus (SYBR Green) Kit (TIANGEN) in a final volume of 20 μL containing 10 μL of PCR buffer, 0.6 μL of each primer (10 μM/μL), 3 μL of template cDNA, and 5.4 μL of DEPC H_2_O and 0.4 μL 50 × ROX Reference Dye. Real-time PCR was performed with SYBR Green using an ABI-7500 thermocycler (Applied Biosystems) and a thermocyling program of 94 °C for 15 min, followed by 40 cycles of 95 °C for 10 s, 60 °C for 32 s. Fluorescence was measured at the end of every 60 °C extension phase. Relative gene expression was calculated according to the Livak method (2^−ΔΔCt^). The experiments were repeated 3 times independently. All the primers used in this study are shown in supplemental Table S2.

## Additional Information

**How to cite this article**: Than, W. *et al*. Analysis of *Sogatella furcifera* proteome that interact with P10 protein of *Southern rice* *black-streaked dwarf virus*. *Sci. Rep.* **6**, 32445; doi: 10.1038/srep32445 (2016).

## Supplementary Material

Supplementary Information

## Figures and Tables

**Table 1:** Putative proteins of *Sogatella furcifera* that interacted with SRBSDV-P10 in a yeast two-hybrid assay.

No.	Putative Protein Name	Hit ACC	E-value
1	AGAP005170-PA	XP_314066.4	1.00E-04
2	AGAP005528-PA	XP_001237680.1	1.00E-66
3	Aldehyde Dehydrogenase	XP_001653928.1	2.00E-07
4	Alpha tubulin 84B	AFX62860.1	0.0
5	Aminomethyltransferase, mitochondrial	KDR19910.1	1.00E-104
6	Atlastin	KDR23965.1	2.00E-91
7	ATP citrate lyase	AAB47486.1	1.00E-150
8	ATP synthase lipid-binding protein, mitochondrial	KDR24087.1	1.00E-38
9	ATPase subunit, putative	BAN20155.1	1.00E-40
10	BCR-associated protein, bap	BAN20871.1	0.002
11	Calcium-transporting ATPase sarcoplasmic/endoplasmic reticulum type	XP_001868895.1	1.00E-76
12	Carboxylesterase	AHI17927.1	0.0
13	Cathepsin L	XP_001842337.1	6.00E-61
14	Cleft lip and palate transmembrane protein 1-like protein	EFN62769.1	5.00E-166
15	Conserved hypothetical protein	XP_001842626.1	3.00E-22
16	Cuticlin-1	KDR07206.1	3.00E-29
17	Cytochrome *b*	YP_008081176.1	4.00E-66
18	Cytochrome B5-like protein	AGM32720.1	2.00E-45
19	Cytochrome *c* oxidase subunit III	AGH29104.1	1.00E-116
20	Delta-9-acyl-CoA desaturase	AHI54575.1	2.00E-131
21	Dolichol-phosphate mannosyltransferase, putative	XP_002429901.1	5.00E-97
22	Dolichyl-diphosphooligosaccharide-protein glycosyltransferase subunit 2	KDQ71487.1	1.00E-45
23	E3 ubiquitin-protein ligase arkadia-B	KDR18887.1	1.00E-05
24	Fatty acid desaturase	AAB17283.1	1.00E-131
25	GG19855	XP_001983129.1	5.00E-177
26	GG13181	XP_001979049.1	1.00E-23
27	GG23285	XP_001970611.1	1.00E-31
28	GL11827	XP_002026631.1	1.00E-07
29	GL21484	XP_002017507.1	3.00E-04
30	Glucosyl glucuronosyl transferases	AGC84403.1	3.00E-41
31	Glutamate [NMDA] receptor-associated protein 1	NP_001040129.2	7.00E-84
32	Glutamate Receptor Gr3	ABD36126.1	2.00E-40
33	Hypothetical protein Aael_Aael006322	XP_001651884.1	0.15
34	Hypothetical protein Aael_Aael012161	XP_001655924.1	2.00E-14
35	Hypothetical protein AND_001938	ETN66288.1	1.00E-38
36	Hypothetical protein Dappudraft_216285	EFX72148.1	3.00E-91
37	Hypothetical protein L798_02550	KDR22066.1	3.00E-28
38	Hypothetical protein Oxytri_13059	EJY66654.1	3.00E-25
39	Hypothetical protein S40285_04320	KFA65580.1	2.00E-63
40	Hypothetical protein Sinv_10231	EFZ14259.1	1.00E-90
41	Hypothetical protein TcasGA2_TC002437	EEZ99680.1	3.00E-11
42	Hypothetical protein Tcasga2_Tc004472	EEZ98861.1	1.00E-09
43	Hypothetical protein Tcasga2_Tc006124	EFA08474.1	4.00E-11
44	Hypothetical protein Trividraft_162453	EHK16550.1	7.00E-73
45	Hypothetical protein X777_00994	EZA57892.1	2.00E-22
46	Hypothetical protein Yqe_06145	ENN77319.1	7.00E-24
47	Hypothetical protein YQE_12924	ENN70419.1	3.00E-13
48	Integral membrane protein 2B	KDR10662.1	4.00E-19
49	Junctophilin	ETN67432.1	2.00E-60
50	l(2)01289 Long form	AAF34751.1	1.00E-89
51	Lactoylglutathione lyase	ETN67394.1	1.00E-27
52	Longwave opsin	BAO03858.1	2.00E-119
53	Mitochondrial Cytochrome C Oxidase Subunit Iv	AGG22607.1	1.00E-57
54	Na+/K+-ATPase beta2	AHH35013.1	1.00E-98
55	NADH dehydrogenase subunit 1	YP_008081177.1	1.00E-120
56	NADH dehydrogenase subunit 4	YP_008081173.1	2.00E-17
57	Nascent polypeptide-associated complex subunit alpha	EZA52213.1	2.00E-73
58	NMDA receptor glutamate-binding chain	BAN21038.1	3.00E-26
59	Phosphoglycerate kinase	ADQ89809.1	7.00E-36
60	Polyubiquitin	NP_001117778.1	0.0
61	28 kDa heat- and acid-stable phosphoprotein-like	XP_003708272.1	2.00E-13
62	Aconitate hydratase, mitochondrial-like	XP_007559829.1	3.00E-70
63	ADP,ATP carrier protein 1	XP_973257.1	1.00E-107
64	Aldehyde dehydrogenase, dimeric NADP-preferring-like	XP_008485684.1	5.00E-26
65	Alkylglycerol monooxygenase	XP_969001.1	4.00E-50
66	B-cell receptor-associated protein 31-like	XP_003707141.1	2.00E-47
67	Calcium-transporting ATPase sarcoplasmic/endoplasmic reticulum type-like	XP_003399858.1	0.0
68	CD63 antigen-like	XP_011145231.1	2.00E-26
69	Cell wall protein DAN4-like	XP_972460.1	2.00E-10
70	Coronin-1C	XP_001950609.1	1.00E-05
71	Crustapain-like	XP_001599980.2	1.00E-13
72	Cytochrome B5	XP_011190618.1	1.00E-18
73	ER membrane protein complex subunit 10-like isoform X2	XP_004927898.1	2.00E-14
74	ER membrane protein complex subunit 4-like isoform 1	XP_395810.4	3.00E-88
75	Eukaryotic translation initiation factor 3 subunit E isoform X2	XP_011176003.1	1.00E-72
76	Growth hormone-inducible transmembrane protein	XP_976102.1	1.00E-90
77	Heterogeneous nuclear ribonucleoprotein Q-like	XP_003402571.1	7.00E-23
78	Integrin beta-3	XP_005311114.1	5.00E-28
79	Lysosome-associated membrane glycoprotein 1	XP_008192998.1	3.00E-05
80	Mitochondrial fission 1 protein	XP_011306233.1	5.00E-51
81	PEST proteolytic signal-containing nuclear protein-like	XP_003704908.1	1.00E-21
82	Plexin domain-containing protein 2-like isoform X3	XP_006559440.1	4.00E-83
83	Polyubiquitin-B isoform X1	XP_010298599.1	2.00E-160
84	Polyubiquitin-B isoform X6	XP_010734822.1	1.00E-138
85	Polyubiquitin-B-like isoform 1	XP_003970845.1	0.0
86	Polyubiquitin-C [Capra hircus]	XP_005691399.1	0.0
87	Proline-rich receptor-like protein kinase PERK14	XP_008636710.1	0.014
88	Protein jagunal-like	XP_003399831.1	2.00E-89
89	Protein lifeguard 4-like	XP_008478082.1	2.00E-38
90	Putative fatty acyl-CoA reductase CG5065	XP_001950244.1	1.00E-12
91	Putative pentatricopeptide repeat-containing protein At5g52630-like isoform X1	XP_006343098.1	1.1
92	RING finger and CHY zinc finger domain-containing protein 1-like	XP_003706619.1	2.00E-43
93	RNA-binding protein 39 isoform X1	XP_008475056.1	2.00E-79
94	Selt-like protein	XP_974477.1	1.00E-67
95	Solute carrier family 35 member B1	XP_008471798.1	1.00E-101
96	Synaptobrevin-like	XP_003400686.1	9.00E-31
97	Translocon-associated protein subunit gamma-like	XP_003705748.1	2.00E-90
98	Predicted: Transmembrane 9 superfamily member 3 isoform X2	XP_008205311.1	8.00E-141
99	U8-agatoxin-Ao1a-like isoform 2	XP_003394078.1	1.00E-21
100	Uncharacterized protein LOC100159027	XP_001951239.1	1.00E-28
101	Uncharacterized protein LOC103506942 isoform X1	XP_008469590.1	9.00E-07
102	Uncharacterized protein LOC656848 isoform X1	XP_008192768.1	2.00E-26
103	Vesicle transport protein SFT2B	XP_975144.1	4.00E-34
104	Vesicle-associated membrane protein-associated protein B	XP_966498.1	2.00E-04
105	Protein TMED8	KDR10690.1	3.00E-76
106	PTPLA domain protein	XP_001861472.1	2.00E-89
107	Putative fatty acyl-CoA reductase	KDR11035.1	1.00E-08
108	Putative fructose-bisphosphate aldolase	ABV60327.1	1.00E-114
109	Putative Mfs-type transporter	KDR11631.1	2.00E-101
110	Putative ubiquitin C variant 2	ACH45553.1	0.0
111	Ribophorin Ii	XP_001841944.1	3.00E-48
112	StAR-related lipid transfer protein 7, mitochondrial	KDR02399.1	2.00E-39
113	Sugar transporter 2	BAI83416.1	2.00E-148
114	Sugar transporter 6	BAI83420.1	2.00E-172
115	Tetraspanin 39D	NP_523612.3	2.00E-18
116	Tetraspanin-18, putative	XP_002427607.1	1.00E-41
117	Titin	KDR21735.1	1.00E-12
118	TPR protein	EKD64441.1	9.1
119	Transmembrane and coiled-coil domains protein 1	KDR19652.1	1.00E-25
120	Tubulin alpha chain, testis-specific	NP_001118163.1	0.0
121	tubulin beta-2c chain	XP_001844630.1	5.00E-90
122	Ubiquitin domain-containing protein 1	KDR08629.1	1.00E-61
123	UBX domain-containing protein, putative	XP_002403277.1	1.00E-05
124	Uncharacterized protein Loc100169264 precursor	NP_001162140.1	1.00E-15
125	Vesicle transport V-SNARE protein Vti1A, putative	XP_002425289.1	2.00E-36
126	Vesicle-associated membrane protein 7	KDR17570.1	2.00E-46
127	Vesicular integral-membrane protein VIP36	EFN72204.1	1.00E-41
128	Vitellogenin	AGJ26477.1	6.00E-169
129	V-type proton ATPase subunit S1	EZA52705.1	8.00E-18
130	Z9-desaturase SFWG5A	AAQ12887.1	2.64E-01

**Table 2:** Gene Ontology (GO) annotation for 28 selected putative proteins of Sogatella furcifera* (MF: molecular function, BP: biological process, CC: cellular component).

No.	Protein name	Hit ACC	E-value	GOs
1	Alpha Tubulin 84b	AFX62860.1	0.0	MF: GTP binding, GTPase activity, structural constituent of cytoskeleton BP: Microtubule-based process, protein polymerization CC: Microtubule
2	Atlastin	KDR23965.1	2.00E-91	MF:GTPase activity, GTP binding
3	ATP citrate lyase	AAB47486.1	1.00E-150	MF: ATP citrate synthase activity, succinate-CoA ligase (ADP-forming) activity BP: Cellular carbohydrate metabolic process CC: Lipid particle
4	ATP synthase lipid-binding protein, mitochondrial	KDR24087.1	1.00E-38	MF: Hydrogen ion transmembrane transporter activity BP: ATP hydrolysis coupled proton transport, ATP synthesis coupled proton transport CC: Integral component of membrane, proton-transporting ATP synthase complex, coupling factor f(0)
5	Calcium- Transporting Atpase Sarcoplasmic/Endoplasmic Reticulum Type	XP_001868895.1	1.00E-76	MF: ATP binding, calcium -transporting ATPase activity, Metal ion binding BP: Calcium ion transmembrane transport, fatty acid beta-oxidation, heart contraction, lipid biosynthetic process, positive regulation of calcium-transporting ATPase activity CC: Endomembrane system, integral component of membrane, nuclear envelope, sarcoplasmic reticulum membrane, endoplasmic reticulum
6	Carboxylesterase	AHI17927.1	0.0	MF: Hydrolase activity
7	Cathepsin L	XP_001842337.1	6.00E-61	MF: Cysteine-type endopeptidase activity BP: Proteolysis
8	Conserved hypothetical protein	XP_001842626.1	3.00E-22	MF: Methyl transferase activity, nucleic acid binding
9	Cuticulin-1	KDR07206.1	3.00E-29	MF: Structural constituent of cuticle CC: Collagen and cuticulin-based cuticle extracellular matrix
10	Cytochrome b	YP_008081176.1	4.00E-66	MF: Electron carrier activity, oxidoreductase activity, metal ion binding BP: Respiratory electron transport chain CC: Integral component of membrane, respiratory chain, mitochondrion
11	Cytochrome *c* oxidase subunit III	AGH29104.1	1.00E-116	MF: Oxidase activity BP: Aerobic electron transport chain CC: Integral component of membrane, mitochondrion
12	Glucosyl glucuronosyl transferases	AGC84403.1	3.00E-41	MF: Transferase activity
13	Integral membrane protein 2B	KDR10662.1	4.00E-19	MF: ATP binding, Beta-amyloid binding BP: Extrinsic apoptotic signaling pathway in absence of ligand, negative regulation of amyloid precursor protein biosynthetic process CC: Endosome membrane, intracellular membrane-bounded organelle membrane
14	Longwave opsin	BAO03858.1	2.00E-119	MF: G-protein coupled receptor activity, photoreceptor activity BP: Phototransduction, visual perception, protein-chromophore linkage CC: Integral component of membrane
15	NADH dehydrogenase subunit 1	YP_008081177.1	1.00E-120	MF: NADH dehydrogenase (ubiquinone) actaivity BP: Mitochondrial electron transport CC: Integral component of membrane, mitochondrion
16	Nascent polypeptide-associated complex subunit alpha	EZA52213.1	2.00E-73	MF: Protein binding BP: Neurogenesis, protein transport
17	Polyubiquitin	NP_001117778.1	0.0	MF: Protein tag BP: Protein ubiquitination CC: Cytosol
18	Coronin-1C	XP_001950609.1	1.00E-05	MF: Actin filament binding BP: Actin cytoskeleton organization CC: Cytoplasm, actin cytoskeleton
19	Growth hormone-inducible transmembrane protein	XP_976102.1	1.00E-90	MF: Protein binding BP: Apoptotic process CC: Integral component of membrane
20	Integrin beta-3	XP_005311114.1	5.00E-28	MF: Fibronectin binding, extracellular matrix binding, protease binding, receptor activity BP: Cell growth, cell migration, protein folding, cell adhesion CC: Plasma membrane, cell surface, filopodium membrane, melanosome, nucleus
21	Sugar transporter 2	BAI83416.1	2.00E-148	MF: Transmembrane transporter activity BP: Carbohydrate transport CC: Integral component of membrane
22	Sugar transporter 6	BAI83420.1	2.00E-172	MF: Substrate-specific transmembrane transporter activity BP: Carbohydrate transport CC: Integral component of membrane
23	Tetraspanin 39 D	NP_523612.3	2.00E-18	MF: Protein kinase activity BP: Protein phosphorylation CC: Integral component of membrane
24	Titin	KDR21735.1	1.00E-12	MF: Actin binding, structural constituent of muscle BP: Cell division, locomotion, muscle attachment, skeletal muscle tissue development
25	Tubulin beta-2C chain	XP_001844630.1	5.00E-90	MF: GTPase activity, GTP binding BP: Microtubule-based process, protein polymerization CC: Microtubule
26	Vesicle transport V-SNARE protein Vti1A	XP_002425289.1	2.00E-36	MF: Binding BP: Intracellular protein transport, vesicle-mediated transport CC: Membrane
27	Vesicle-associated membrane protein 7	KDR17570.1	2.00E-46	MF: SNARE binding activity BP: Vesicle-mediated transport CC: Integral component of membrane
28	Vitellogenin	AGJ26477.1	6E-169	MF: Lipid transporter activity BP: Lipid transport

## References

[b1] Zhou G. . Southern rice black-streaked dwarf virus: a new proposed Fijivirus species in the family Reoviridae. Chin. Sci. Bull. 53, 3677–3685 (2008).

[b2] Zhang P. . Simultaneous detection and differentiation of Rice black streaked dwarf virus (RBSDV) and Southern rice black streaked dwarf virus (SRBSDV) by duplex real time RT-PCR. Virol. J. 10, 24–35 (2013). 23331990 10.1186/1743-422X-10-24 PMC3610162

[b3] Hoang A. T. . Identification, Characterization, and Distribution of Southern rice black-streaked dwarf virus in Vietnam. Plant Dis. 95, 1063–1069 (2011). 10.1094/PDIS-07-10-0535 30732067

[b4] Zhou G. . Southern rice black-streaked dwarf virus: a white-backed planthopper-transmitted fijivirus threatening rice production in Asia. Front. Microbiol. 4, 270–294 (2013). 24058362 10.3389/fmicb.2013.00270 PMC3766826

[b5] Yang C. Y. & Frohman M. A. Mitochondria: signaling with phosphatidic acid. Int. J. Biochem. Cell B. 44, 1346–1350 (2012). 10.1016/j.biocel.2012.05.006 PMC3380155 22609101

[b6] Cuong H. V. . Rice dwarf disease in north Vietnam in 2009 is caused by southern rice black-streaked dwarf virus (SRBSDV). Bull. Inst. Trop. Agr., Kyushu Univ. 32, 85–92 (2009).

[b7] Matsukura K. . Dynamics of Southern rice black-streaked dwarf virus in rice and implication for virus acquisition. Virology 103, 509–512 (2013). 10.1094/PHYTO-10-12-0261-R 23301813

[b8] Wang Q. . The complete genome sequence of two isolates of Southern rice black-streaked dwarf virus, a new member of the genus Fijivirus. J. Phytopathol. 158, 733–737 (2010).

[b9] Pu L. . Transmission characteristics of Southern rice black-streaked dwarf virus by rice planthoppers. Crop Prot. 41, 71–76 (2012).

[b10] Tu Z. . Effects of southern rice black-streaked dwarf virus on the development and fecundity of its vector, Sogatella furcifera. Virol. J. 10, 145–152 (2013). 23663428 10.1186/1743-422X-10-145 PMC3698214

[b11] Dupo A. L. B. & Barrion A. T. Taxonomy and general biology of delphacid planthoppers in rice agroecosystems In: Planthoppers: new threats to the sustainability of intensive rice production systems in Asia (eds Heong K. L. & Hardy B.) Los Baños (Philippines). International Rice Research Institute, 3–155 (2009).

[b12] Zhang H. M. . A black-streaked dwarf disease on rice in China is caused by a novel fijivirus. Arch. Virol. 153, 1893–1898 (2008). 18820828 10.1007/s00705-008-0209-4

[b13] Callaway A. . The multifunctional capsid proteins of plant RNA viruses. Annu. Rev. Phytopathol. 39, 419–460 (2001) 11701872 10.1146/annurev.phyto.39.1.419

[b14] Maruyama W. . The minor outer capsid protein P2 of rice gall dwarf virus has a primary structure conserved with, yet is chemically dissimilar to, rice dwarf virus P2, a protein associated with virus infectivity. Arch. Virol. 142, 2011–2019 (1997). 9413509 10.1007/s007050050218

[b15] Toshihiro O. & Jin Y. Role of outer capsid proteins in transmission of Phytoreovirus by insect vectors. Adv. Virus Res. 54, 15–43 (1999). 10547673 10.1016/s0065-3527(08)60364-4

[b16] Huo Y. . Transovarial transmission of a plant virus is mediated by vitellogenin of its insect vector. PLoS Pathog. 10, e1003949 (2014). 24603905 10.1371/journal.ppat.1003949 PMC3946389

[b17] Power A. G. Insect transmission of plant viruses: a constraint on virus variability. Curr. Opin. Plant Biol. 3, 336–340 (2000). 10873852 10.1016/s1369-5266(00)00090-x

[b18] Hogenhout S. A., Ammar E.-D., Whitfield A. E. & Redinbaugh M. G. Insect vector interactions with persistently transmitted viruses. Annu. Rev.Phytopathol. 46, 327–359 (2008). 18680428 10.1146/annurev.phyto.022508.092135

[b19] Hajano J. U. D. . Quantification of southern rice black streaked dwarf virus and rice black streaked dwarf virus in the organs of their vector and nonvector insect over time. Virus Res. 208, 146–155 (2015). 26116274 10.1016/j.virusres.2015.06.015

[b20] Citovsky V. . Subcellular localization of interacting proteins by bimolecular fluorescence complementation in planta. J. Mol. Biol. 362, 1120–1131 (2006). 16949607 10.1016/j.jmb.2006.08.017

[b21] Wang Y. . Localization and distribution of *Wheat dwarf virus* in its vector leafhopper, *Psammotettix alienus*. Phytopathology 104, 897–904 (2014) 24502202 10.1094/PHYTO-09-13-0251-R

[b22] Liu B. . Differential proteomics profiling of the ova between healthy and *Rice stripe virus*-infected female insects of *Laodelphax striatellus*. Sci. Rep. 6, 27216 (2016). 27277140 10.1038/srep27216 PMC4899684

[b23] Mar T. . Proteomic analysis of interaction between P7-1 of Southern rice black-streaked dwarf virus and the insect vector reveals diverse insect proteins involved in successful transmission. J. Proteomics 102, 83–97 (2014). 24650428 10.1016/j.jprot.2014.03.004

[b24] Feng D. . Ultrastructural localization of vesicle-associated membrane protein(s) to specialized membrane structures in human pericytes, vascular smooth muscle cells, endothelial cells, neutrophils, and eosinophils. J. Histochem. Cytochem. 49, 293–304 (2001). 11181732 10.1177/002215540104900303

[b25] Galli T. . A novel tetanus neurotoxin-insensitive vesicle associated membrane protein in SNARE complexes of the apical plasma membrane of epithelial cells. Mol. Biol. Cell 9, 1437–1448 (1998). 9614185 10.1091/mbc.9.6.1437 PMC25366

[b26] Coco S. . Subcellular localization of tetanus neurotoxin-insensitive vesicle associated membrane protein (VAMP)/VAMP7 in neuronal cells: evidence for a novel membrane compartment. J. Neurosci. 19, 9803–9812 (1999). 10559389 10.1523/JNEUROSCI.19-22-09803.1999 PMC6782963

[b27] Advani R. J. . VAMP-7 mediates vesicular transport from endosomes to lysosomes. J. Cell Biol. 146, 765–775 (1999). 10459012 10.1083/jcb.146.4.765 PMC2156136

[b28] Pryor P. R. . Combinatorial SNARE complexes with VAMP7 or VAMP8 define different late endocytic fusion events. EMBO Rep. 5, 590–595 (2004). 15133481 10.1038/sj.embor.7400150 PMC1299070

[b29] Rao S. K. . Identification of SNAREs involved in synaptotagmin VII-regulated lysosomal exocytosis. J. Biol. Chem. 279, 20471–20479 (2004). 14993220 10.1074/jbc.M400798200

[b30] Steffen A. . MT1-MMP-dependent invasion is regulated by TI-VAMP/VAMP7. Curr. Biol. 18, 926–931 (2008). 18571410 10.1016/j.cub.2008.05.044

[b31] Kloepper T. H. . SNAREing the basis of multicellularity: consequences of protein family expansion during evolution. Mol. Bio.l Evol. 25, 2055–2068 (2008). 10.1093/molbev/msn151 18621745

[b32] Flowerdew S. E. & Burgoyne R. D. A VAMP7/Vti1a SNARE complex distinguishes a non-conventional traffic route to the cell surface used by KChIP1 and Kv4 potassium channels. Biochem. J. 418, 529–540 (2009). 19138172 10.1042/BJ20081736 PMC2650881

[b33] Villarreal A. M. . Molecular characterization and functional significance of the Vti family of SNARE proteins in tick salivary glands. Insect Biochem. Molec. 43, 483–493 (2013). 10.1016/j.ibmb.2013.03.003 PMC3633690 23499931

[b34] Rojas-Rivera D. & Hetz C. TMBIM protein family: ancestral regulators of cell death. Oncogene 34, 269–280 (2014). 24561528 10.1038/onc.2014.6

[b35] Oka T. . Identification of a novel protein MICS1 that is involved in maintenance of mitochondrial morphology and apoptotic release of cytochrome c. Mol. Biol. Cell 19, 2597–2608 (2008). 18417609 10.1091/mbc.E07-12-1205 PMC2397309

[b36] Lisak D. A. . The transmembrane Bax inhibitor motif (TMBIM) containing protein family: Tissue expression, intracellular localization and effects on the ER CA^2+^-filling state. BBA-Mol. Cell Res. 1853 2104–2114 (2015). 10.1016/j.bbamcr.2015.03.002 25764978

[b37] Zhou J. . Comparative genomics and function analysis on BI1 family. Comput. Biol. Chem. 32, 159–162 (2008). 18440869 10.1016/j.compbiolchem.2008.01.002

[b38] Beatrix B. The alpha and beta subunit of the nascent polypeptide-associated complex have distinct functions. J. Biol. Chem. 275, 37838–37845 (2000). 10982809 10.1074/jbc.M006368200

[b39] Pech M. . Dual binding mode of the nascent polypeptide-associated complex reveals a novel universal adapter site on the ribosome. J. Biol. Chem. 285, 19679–19687 (2010). 20410297 10.1074/jbc.M109.092536 PMC2885246

[b40] Kirstein-Miles J. . The nascent polypeptide-associated complex is a key regulator of proteostasis. EMBO J. 32, 1451–1468 (2013). 23604074 10.1038/emboj.2013.87 PMC3655472

[b41] Ackerman S. H. & Tzagoloff A. Function, structure, and biogenesis of mitochondrial ATP Synthase. Prog. Nucleic. Acid Re. 80, 95–133 (2005). 10.1016/S0079-6603(05)80003-0 16164973

[b42] Shan Y. . Developments of combretastatin A-4 derivatives as anticancer agents. Curr. Med. Chem. 18, 523–538 (2011). 21143124 10.2174/092986711794480221

[b43] McKean P. G. . The extended tubulin superfamily. J. Cell Sci. 114, 2723–2733 (2001). 11683407 10.1242/jcs.114.15.2723

[b44] Oakley B. R. An abundance of tubulins. Trends Cell Biol. 10, 537–542 (2000). 11121746 10.1016/s0962-8924(00)01857-2

[b45] Mueckler M. Facilitative glucose transporters. Eur. J. Biochem. 219, 713–725 (1994). 8112322 10.1111/j.1432-1033.1994.tb18550.x

[b46] Bailey R. L. . The emerging role of tetraspanin microdomains on endothelial cells. Biochem. Soc. T. 39, 1667–1673 (2011). 10.1042/BST20110745 22103505

[b47] Hutchens J. . Structurally similar drosophila a-tubulins are functionally distinct *in vivo*. Mol. Biol. Cell 8, 481–500 (1997). 9188100 10.1091/mbc.8.3.481 PMC276099

[b48] Bian X. . Structures of the atlastin GTPase provide insight into homotypic fusion of endoplasmic reticulum membranes. P. Natl. Acad. Sci. USA 108, 3976–3981 (2011). 10.1073/pnas.1101643108 PMC3054032 21368113

[b49] Howell N. Evolutionary conservation of protein regions in the proton motive cytochrome b and their possible roles in redox catalysis. J. Mol. Evol. 29, 157–169 (1989). 2509716 10.1007/BF02100114

[b50] Esposti M. D. . Mitochondrial cytochrome b: evolution and structure of the protein. Biochim. Biophys. Acta 1143, 243–271 (1993). 8329437 10.1016/0005-2728(93)90197-n

[b51] Yusnita Y. . Mutations in mitochondrial NADH dehydrogenase subunit 1 (mtND1) gene in colorectal carcinoma. Malaysian J. Pathol. 32, 103–110 (2010). 21329181

[b52] Kramer J. M. . The sqt-1 gene of C. elegans encodes a collagen critical for organismal morphogenesis. Cell 55, 555–565 (1988). 3180220 10.1016/0092-8674(88)90214-0

[b53] Andersen S. O. . Insect cuticular proteins. Insect Biochem. Molec. 25, 153–176 (1995). 10.1016/0965-1748(94)00052-j 7711748

[b54] Cilia M. . Genetics coupled to quantitative intact proteomics links heritable aphid and endosymbiont protein expression to circulative polerovirus transmission. J. Virol. 85, 2148–2166 (2010). 21159868 10.1128/JVI.01504-10 PMC3067806

[b55] Liu W. . Proteomic analysis of interaction between a plant virus and its vector insect reveals new functions of hemipteran cuticular protein. Mol. Cell. Proteomics 14, 2229–2242 (2015). 26091699 10.1074/mcp.M114.046763 PMC4528249

[b56] Wang H. . Integrative proteomics to understand the transmission mechanism of Barley yellow dwarf virus-GPV by its insect vector *Rhopalosiphum padi*. Sci. Rep. 5, 10971 (2015). 26161807 10.1038/srep10971 PMC4498328

[b57] Pickart C. M. Mechanisms underlying Ubiquitination. Annu. Rev. Biochem. 70, 503–533 (2001). 11395416 10.1146/annurev.biochem.70.1.503

[b58] Gao G. & Luo H. The ubiquitin–proteasome pathway in viral infections. Can. J. Physiol. Pharm. 84, 5–14 (2006). 10.1139/y05-144 16845885

[b59] Boulant S. . Dynamics of virus-receptor interactions in virus binding, signaling, and endocytosis. Viruses 7, 2794–2815 (2015). 26043381 10.3390/v7062747 PMC4488714

[b60] Hajano J. U. D. . Screening of rice (*Oryza sativa*) cultivars for resistance to rice black streaked dwarf virus using quantitative PCR and visual disease assessment. Plant Pathol. 10.1111/ppa.12534 (2016).

