# Supplementary material for: Analysis of Sogatella furcifera proteome that interact with P10 protein of Southern rice black-streaked dwarf virus
Source: Sci Rep. 2016 Sep 22;6:32445. doi: 10.1038/srep32445 (PMC5032029; doi:10.1038/srep32445)
Supplement: Supplementary Information [file srep32445-s1.pdf]

Supporting Information for

Analysis of *Sogatella furcifera* proteome that interact with P10 protein of *Southern rice black-streaked dwarf virus*

Win Than*, Faliang Qin*, Wenwen Liu, Xifeng Wang**

State Key Laboratory for Biology of Plant Diseases and Insect Pests, Institute of Plant Protection, Chinese Academy of Agricultural Sciences, Beijing 100193, China

*These authors contributed equally to this work.

**Corresponding author: xfwang@ippcaas.cn

Supplemental Table-S1: Complete nucleotide sequence for each gene

Name	Complete nucleotide sequence
**Vesicle-associated membrane protein 7**<br>5' untranslated region [32 bp]<br>CDS (33 bp-683 bp) [651 bp]<br>3' untranslated region [693 bp]<br>Poly (A) tail [29 bp]<br>Total [1405 bp]	atctcactttcatttgattaaatcgaataaaa**atg**cttatcctatacagtgtagttgcaagagggaccacggttctggcaaaatttgcaacatgcgctggaaatttttccgaggtgacagaacaaatcctggcgaaaattggtccagaaaacaggaggaagactctctctcacagtagctatttattccattacatatgtgaggacagaattgtttacatgtgtattgcggatgatgagtttgaacgaccaagagcgtttttatacttgaatgaaataaaaaagaggttccgttcaacatatggcagcagagctgacacggccattgcttatgccatgaactcagagtttggccctattttaggaaatgaaatgaaatactattcagaatcgaaggatattgacacaatttcacgagtacatggcgaattggatgaattgaaagatataatggttcacaatattgataacattgctatgagagatgaacgcctcgaattgctggttgataaaacagaaaatctaacagccagctctgtcacattccgagcaactagtagaaatttacagcgagctctcttctggaaaaatatgaaactctatatgataatctcagctattgttttggtaataatttatttcatcttagctatgtcttgtggatcgactttgtccagttgc**tga**aagactacagagtaacaatcacctgtacggtttaattataatccgatgtattcttcagtaaagagtcttccttgtgatatgttcaatgataaatttatactattcttctaaagtagcatgtaaaacataagctacggtactgtatttttttattttatccttatttcacggttcaattaatcaccagaaattattttgaataatccaatcatattcttaattattatatccataaacaaatggtaatgtgactgtatccactgtattaaataatagattgcaataaatttcaattaagagtttttttactgcagtttgagtaatacaaaatagatccgttttatattagttgtttatatttattcacagattgttttattgtcagagaagtgaaacattgtatttgcataaggaatgtatgatagatacctatgaagatattattttgaattattctattttcttgataaatattatgttcataaacaaagggaaatgtaaatgtattgtaatccactgttttagatcatagattgtaataaatttctattgaagtacggtttttttagattgcagtttcagtaatacaaaatagaaccgtttttttattagttgcataaatttgaaaatatatatttctataaaaaatattgttttattatcggagaattgaaacaatttatttgcataatgaaaataaaaaaaaaaaaaaaaaaaaaaaaaaaaa
**Vesicle transport V-SNARE protein Vti1a**<br>5' untranslated region [54 bp]<br>CDS (55 bp-723 bp) [669 bp]<br>3' untranslated region [952 bp]<br>Poly (A) tail [30 bp]<br>Total [1705 bp]	aatgaaaaattattaataagttcaaactaatttagtagcctaaaacattagatt**atg**gctgctttaattgatgtttatgagcagcaatatgcagtcatcacagcagatgttacttcaaagattggaagattatcatctctttctggaggagaacgacgtcatgttatttcggaattggataagctgtttgatgaagccaaagagcttatggaacaaatgggccttgaagttcacgagttgaaatcagcagatcaatcaaaacttaagaacagaattgaaagttataaggcagaactgaaaagactagaacaagaatttactaatgctaaaaagaatatcaactcacaaaatggctacagcgataggatagaactgtatagcgaaagtataagcgtcaatgaggaacagaagcaaaggcttttggacaatgctgaaatggttgagcgcactggcaagaaattgactgctggctatcaagtgcttttggagactgaagatattggcaatcaagttctcagggacttgcattcgcagagggaaacgatacagaaatcgaggtctaggttaagagaaacaaacgccgagctgggaagaagtaacagaatcatcaattcgatgatcaacagatctctgcaacacaggttcatcttgacagctatcgccatcgtgttctgccttgtggtcgtgatcagcatctacatatcagctactcgctctcga**taa**actgcacttgtcaccatttagtactctactcggtgtatttcctaatcattctataattaaataatttattcactgctctagtatgaaattaaactaactatcattgaattgaactatcactgttgaaacaaacacctagtaaattattaattgtgtaaaagacaaaggacttcaatagctgtctgcctctctccgtcttaatataaaatgatctaataaaggattatctcatgctcaatatatgggtatcgtatgtgactatgattcttgatatcagtatttgaaaatgcattcatattttcctagaataataacttacgtctatcattgcacagtatattgtactaatttcataactcacatcatattgtttatcattttagatattttaaagtctgattgtgacttaattgtaacgaagatcattctggagctataaaatacttgagttattccaatatcttaattgctattgcaagaaaatgttatttgttttgtatgctgaatctattaaacgatagaaatctatgaaaagtttggat

	gctttttgtgatgtaaataaatagtaaatagtaagctagagtaataatcaattatcttgaaattaatacttgagtttcaaataaggatggatagtatatgcgtaagt<br>atggctagatggatgataatagatggatagtatggctagtaagtttttttgttttatgttgtcctatacaaaatcttaattaattgatatctgattggaaactgctaattt<br>tattctatataccctcgtattgattcatgatgcaaatattttccaattattatactgtaagttactcatattctacttcacatgtctgttgaaaaatttgagaattgtccat<br>tttaatgtctttttgtgagttgtaaatatttcatgtgtaacttattattgtaaataaaataaatgtctcatttattgttcgaaaaaaaaaaaaaaaaaaaaaaaaaaaa<br>aa
**Growth hormone-inducible transmembrane protein**<br>5' untranslated region [57 bp]<br>CDS (58 bp-1083 bp) [1026 bp]<br>3' untranslated region [297 bp]<br>Poly (A) tail [30 bp]<br>Total [1410 bp]	tcatctaggcctactgtcagctatttggaatacactttctagaactaactcttgatg**atg**ttggcctcacgactttgtagcttgcacctagcaccactcggaagg<br>acatctattatccgagtggtgcaaaacagtaagctgccttcgacaaaaagcacaagcaatgaactgtttcgactctacagcaaccagggcagaggatcatg<br>gtctagggcatctcctcgtagacgtaccatcaaggaaatcgccatgcaaccagcatctggcacagctttcaacgttggcacagcagcagtggcgggcgg<br>agcagctgtcggaatcggtgcgttgtgcttctacggattgggcttggcatccaaacctggcgccatggatcagtcaatgatgtggcccgagtatgtgaagg<br>aaagaatcagatcaacctatatgtattttggatcatcagtggctctcacagcagccagtgcttacgcttgcttcaattcaccagtcatggtcaaattgatgatga<br>agaactctttaatggcaattgcattgagtttggctgcgatgattggtacatcggttattgctaggagcattccttacacagaaggctttggattgaagcaggcc<br>gcctgggctctgcacaccagtgtcataggagcagtcatcgcgcccatctgtttcgtaggaggccccatcctagttagagctgcttggtacacggccggcgt<br>agtgggtgggctgtcgttggtggctgcttgcgcacccagcgaacagttcctgaacatggcggggccgctcagcatgggcctggcggtagtgtttgccgc<br>ctccctcggctctgtctttatcgcccccaccaccgcactcggctccggactctactccatcagcctttacggcggcctgctcctattctccggatttctgcttta<br>cgacacgcagaagatcatcaagagtgccgaaactcattcgccttacgccatggagaaatacgatccgatcaacatgtctatgggcatctacatggatacaa<br>tcaacatcttcataagaattgctacaattttgaacaatggtggctcatcaaaaaggaga**taa**gtcaatttagaactgattatcctagaaaattaatgtaaactata<br>agaattcatcattttcatctgccaatttcgtcaaaatatttattttagttctgatcatggttgacataaagcaatgtattatactcctttcaacaaaaatttgtaagctta<br>ttaatcaaatgatttgatggaaaaattgtattatacttaatttttcacaataaattgtaagattaaaatagtttgaaatacgaattactgttccaaattattgtaaaaact<br>tcatccacaataaatcagttcatttgaaccgaaaaaaaaaaaaaaaaaaaaaaaaaaaaaa
**Nascent polypeptide-associated complex subunit alpha**<br>5' untranslated region [24 bp]<br>CDS (25 bp-666 bp) [642 bp]<br>3' untranslated region [1517 bp]<br>Poly (A) tail [30 bp]<br>Total [2213 bp]	ctgaaatcttccattttcataaaa**atg**ccagaagcaacagaacttgataaaccagcccccactttgacggaagttaaaaatgatgcatcagggacggagtca<br>gattccgacgactcaattcctgagctggaggagggaccaaaaggagcctccgatggtccaggatcatccattgcgtttggggaaggagctcctattgatat<br>ggtcagcaaagccaaacaatctcgtggagaaaagaaggctcgaaaaattatgttgaagctgggcctcaaaccgatcccaggagtctcaagagtgacaat<br>ccgcaaatccaagaatattctcttcgtaatcaacaaaccagacgttttcgaaaatcctgcatccgatacctacattgttttcggtgaagccaagattgaagatct<br>tggacaacaagcgcaagttgctgcagccgaaaaattcaaagcccctgaaatttccgcagctgcagaggctggaggtagtgctgcgttggtgacacctata<br>caagaggaaagcgacgaagaagtcgatgagaccggcgttgaagacaaggatgttgatttggtgatgtctcaggcgaacgtcagtcgaggaaaagcaat<br>caaggctcttcgaaacaataagaacgacatcgttaatgcaatcatggaactcaccata**tga**agtgtttggatccgagtcgaagtgtcggtatcagggttttca<br>attttatcgagtgtttttcatctttacttatgatgtttgatttatttctgttgtgtaaaagacttctatcgtaatactatttcacttaattctctcatcaaagaaaaatcttac<br>gaattttaaaagcgtgtaccaagaaatagttttgctgtttttgttgaataatagaacatgttatcggtgtagccaatatactaaaacacgtattacatctgaggtat

	gatttatttttgttcaatatttatatgattacattatttttacttgatttctcatcaagttgaaaattcagtaatttcaaaagcatgtactaagaaaatattttccattctctg<br>tctagaaataaaataagcgttatcgttgatgttgaggcttagtatacttcaacatcttttacaccttaactttgatttttttaaaaatacttttatgatcataggctacta<br>tttttacgtaatctctcatcaagaaaaaaataagtcacgtttttaaaagcgagaacttggataagttttggagttctttgtttagttttgcagttcaaattgtaaaatg<br>agttatcagtatagcctagtagatttgaagattgcattttggtctaggatcgctgttgtggtcacgggttggattctcattcctcttggaattccggagcctcctca<br>gaccaggaggtatctggtcaatcgaatgtatgtgaaatgttacagaagtattcatttcttgctttaccatagtttaattatttgaaaataggcctaccctacctgta<br>gataattttgttttaaagtgtagcagcttcgtgaatcaacctcactcagttatattattcacatgtcccattctgttatacttttgtctgtgggtgatgcccacatggg<br>ctccttggcctgtgtgtgggtaatgagagggatgatgatgagagatgacgactacttttgtagcagtctggaccgacggcttatcgtcttctcctaaatatggt<br>catctgaatattttttaatcatttaatgatcttacttcaatctcaaatttcatagttcgatttttttatttttaaaagttcttcttcttcttcttcttcttcttcttcttcttctt<br>cttcttcttcttcttcgatggccctacagctcaaatcgagccctggcctcctcagcctgaggctgaagtccaaattactctgttaaaatttagaatttaactctgtt<br>gaaccaattgttatgccaacaggcattattcatagtattcttttaagattctctgtatagtagaatccaaatatagaatgggaatcgaacccaactattagattaaa<br>gttgtgagccctgtctactgggttatactagattattttatttctagtactgcgttttcaattattacaatttgtatattattgttattcatgttgataactataatttgtaag<br>agtgaactatagttttaataataaatcattttcgttcttgctttaaaaaaaaaaaaaaaaaaaaaaaaaaaaaa
**ATP synthase lipid-binding protein**<br>5' untranslated region [46 bp]<br>CDS (47-469 bp) [423 bp]<br>3' untranslated region [598 bp]<br>Poly (A) tail [30 bp]<br>Total [1097 bp]	ggagttcgtcgctgctgtgcaagaaaacccaactccaaactccaaa**atg**tactcaaacattgcaagaattgttggcccagctgcccgatctgctttggtatc<br>aggatcaagagcctacgtgaggccactgagcagttcagtgtgcacacccaccgctttcaactcacagaatacacagagcactccaaccgttagtttgttac<br>ccgccgtaaggaacttccaaacctcagctgtgtcgcgcgatattgactcagctgccaaattcatcggtgccggagccgccaccgttggagtcgctggctc<br>aggagccggaatcggaacagtgttcggttcgctgatcatcggctacgccaggaatccatcgctcaagcagcagctgttctcttacgccattctgggattcg<br>ccctgtccgaggccatgggtctcttctgtcttatgatggccttcctgctcttgttcgctttc**taa**acagcatttctacatttagttgattacaaaaataagataatta<br>taaattgaaaaacagaacaggactacactgccacttggggtgatttaaaaccaggtcgcgatcattttcatttaaaaagggttgttggtagcgaggagtggtt<br>acggcgaacgatcgaggctcgatattcatcgactatcgatatttatcgactagtgttggggcttgcacattagtggttgaatatcgaacgatgtttggtttaatt<br>ggggcaaaccttccatcgaaactcttacaccaaaacatcggctcaccacggtgtaaacactcctagattttaagtttcatccctccttcagcaataataagctt<br>acaagctagatgaagaaacaattcaccatcaatgtttatttatgttactagttgtacttctcaaaaaatttaaataattgtaatttttcatccatctatccgggatga<br>gttgttgacattacaatgctattgaacttcaatttttgttataaaaaatcggctttattatcgtcttaattgtttcttttgaaacttggatgatatgtcagcgagagaca<br>tttccattgtgtacagtgttagagtaaaaaaaaaaaaaaaaaaaaaaaaaaaaaa

Supplemental Table S2: Primers used in this study

Primer name	Primer sequence
**Yeast two-hybrid**	
SRBSDV-P10-F:	5'- ATTAACAAGGCCATTACGGCCATGGCTGACATAAGACTTGA-3'
SRBSDV-P10-R:	5'-AACTGATTGGCCGAGGCGGCCCCTCTGGTGACTTTATTTAA-3'
pPR3N-F	5'-GTCGAAAATTCAAGACAAGG3'
pPR3N-R	5'-AAGCGTGACATAACTAATTAC3'
M13-F	5'-CGCCAGGGTTTTCCCAGTCACGAC-3'
M13-R	5'-AGCGGATAACAATTTCACACAGGA-3'
**Full-length gene sequence amplification**	
**Vesicle-associated membrane protein 7**	
GSP-1	5'-AACCGTACAGGTGATT-3'
GSP-2	5'-TAGTTGCTCGGAATGTGA-3'
GSP-3	5'-ATCCAATTCGCCATGTAC-3'
**Vesicle transport V-SNARE protein Vti1a**	
GSP-1	5'- ATCACGACCACAAGG-3'
GSP-2	5'-GATTTCTGTATCGTTTCCCTCT-3'
GSP-3	5'-GATAGCCAGCAGTCAATTTCTT-3'
**Growth hormone-inducible transmembrane protein**	
GSP-1	5'-TAAAGGCTGATGGAGTA-3'
GSP-2	5'-CAACGACAGCCCACCCACTA-3'
GSP-3	5'-CCAATCATCGCAGCCAAACT-3'

**Nascent polypeptide-associated complex subunit alpha**	
GSP-1	5'-TTCTTCGTCGCTTTCC-3'
GSP-2	5'-ATCTTCAATCTTGGCTTCACC-3'
GSP-3	5'-ATTCTTGGATTTGCGGATTG-3'
**ATP synthase lipid-binding protein**	
GSP-1	5'-GCTTGAGCGATGGATT-3'
GSP-2	5'- TACGGCGGGTAACAAACT-3'
GSP-3	5'-GGCGGGTAACAAACTAACG-3'
**GST Pull-down assay**	
pGEX+P10–F	5'-CGCGGATCCATGGCTGACATAAGACTTGA-3'
pGEX+P10–R	5'-CCGCTCGAGCATCTGGTGACTTTATTTAA-3'
pCold+VAMP7-F	5'-CGCGGATCCATGCTTATCCTATACAGTGTA -3'
pCold+VAMP7-R	5'-CCCAAGCTTTCAGCAACTGGACAAAGTCGA-3'
pCold+Vti1a-F	5'-CGCGGATCCATGGCTGCTTTAATTGATGTTTA-3'
pCold+Vti1a-R	5'-CCCAAGCTTTTATCGAGAGCGAGTAGCTGATA-3'
pCold+Ghitm-F	5'-CGCGGATCCATGTTGGCCTCACGACTTTGTA-3'
pCold+Ghitm-R	5'-CCCAAGCTTTTATCTCCTTTTTGATGAGCCA-3'
pCold+ Nascent-F	5'-CGCGGATCCATGCCAGAAGCAACAGAACTTGA-3'
pCold+ Nascent-R	5'-CCCAAGCTTTCATATGGTGAGTTCCATGA-3'
pCold+ ATPase-F	5' -CGCGGATCCATGTACTCAAACATTGCAAG-3'
pCold+ATPase-R	5'-CCCAAGCTTTAGAAAGCGAACAAGAGCAG-3'
**Gene expression analysis (RT-qPCR)**	
qVAMP-7-F	5'-ACAGAACAAATCCTGGCGAAAA-3'
qVAMP-7-R	5'-CAAACTCATCATCCGCAATACA-3'
q Vti1a-F	5'-CGCAGAGGGAAACGATACA-3'

q Vti1a-R	5'-ACCACAAGGCAGAACACGA-3'
q Ghitm-F	5'-GTAGTGGGTGGGCTGTCGTT-3'
q Ghitm-R	5'-GCCGTAAAGGCTGATGGAGT-3'

Supplementary Fig.1: GST Pull-down analysis of the interaction between SRBSDV-P10 and WBPH protein by Western blot

(A)

1: Protein Ladder

2: VAMP7-His (39.7 kDa) (Input)

3: VAMP7-His + GST (Pull-down = Negative control)

4: VAMP7-His + P10-GST (Pull-down)

(VAMP7-His = 39.7 kDa)

5: Protein Ladder

6: Vti1A-His (39.7 kDa) (Input)

7: Vti1A-His + GST (Pull-down = Negative control)

8: Vti1A-His + P10-GST (Pull-down)

(Vti1A -His = 40.4 kDa)

9: Protein Ladder

10: Ghitm-His (51 kDa) (Input)

11: Ghitm-His + GST (Pull-down = Negative control)

12: Ghitm-His + P-10-GST (Pull-down)

(Ghitm-His = 51 kDa)

(B)

Lane

1: Protein Ladder

2: Nascent polypeptide-associated complex subunit alpha-His (37.5 kDa) (Input)

3: Nascent polypeptide-associated complex subunit alpha-His + GST (Pull-down = Negative control)

4: Nascent polypeptide-associated complex subunit alpha-His + P10-GST (Pull-down)

5: Protein Ladder

6: ATP synthase lipid-binding protein-His (29.4 kDa) (Input)

7: ATP synthase lipid-binding protein-His + GST (Pull-down = Negative control)

8: ATP synthase lipid-binding protein-His + P10-GST (Pull-down)

A

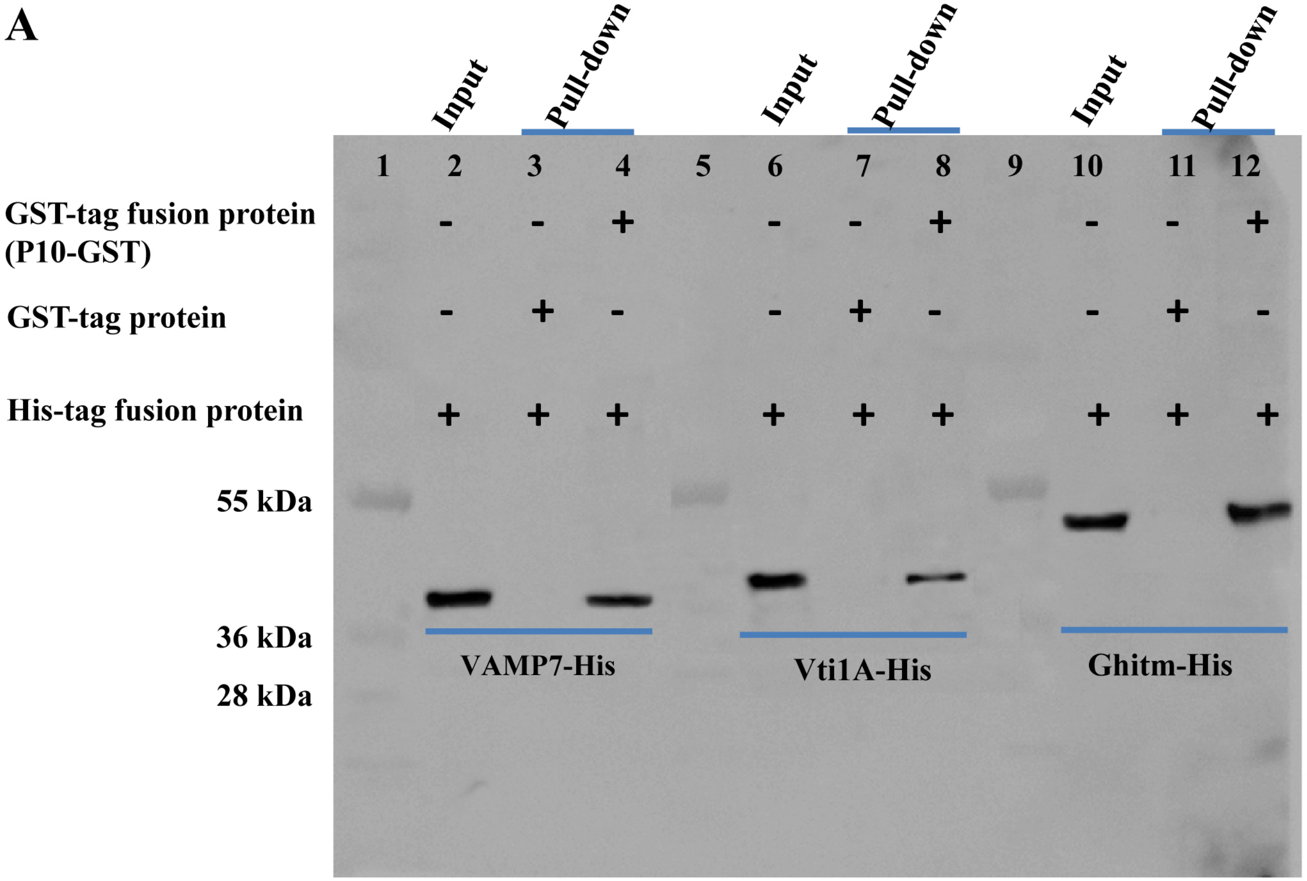
Input
Pull-down
Input
Pull-down
Input
Pull-down
1 2 3 4 5 6 7 8 9 10 11 12
GST-tag fusion protein (P10-GST)
GST-tag protein
His-tag fusion protein
55 kDa
36 kDa
28 kDa
VAMP7-His
Vti1A-His
Ghitm-His

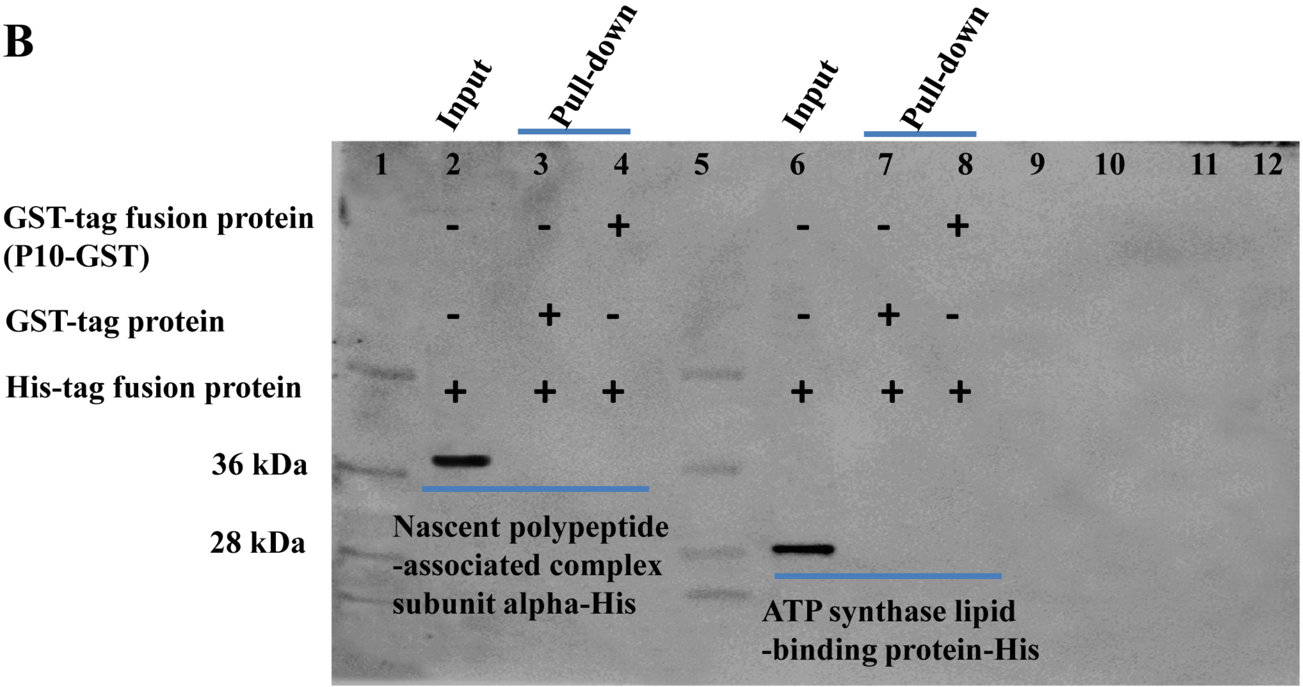
B
Input
Pull-down
Input
Pull-down
1 2 3 4 5 6 7 8 9 10 11 12
GST-tag fusion protein (P10-GST)
- - + - - +
GST-tag protein
- + - - + -
His-tag fusion protein
+ + + + + +
36 kDa
28 kDa
Nascent polypeptide -associated complex subunit alpha-His
ATP synthase lipid -binding protein-His
